# Sequential stages and distribution patterns of aging-related tau astrogliopathy (ARTAG) in the human brain

**DOI:** 10.1186/s40478-018-0552-y

**Published:** 2018-06-11

**Authors:** Gabor G. Kovacs, Sharon X. Xie, John L. Robinson, Edward B. Lee, Douglas H. Smith, Theresa Schuck, Virginia M.-Y. Lee, John Q. Trojanowski

**Affiliations:** 1 Institute of Neurology, Medical University of Vienna, AKH 4J, Währinger Gürtel 18-20, 1097 Vienna, Austria; 2 Center for Neurodegenerative Disease Research (CNDR), Institute on Aging and Department of Pathology & Laboratory Medicine, Perelman School of Medicine (PSOM) at the University of Pennsylvania, HUP Maloney 3rd Floor, 36th and Spruce Street, Philadelphia, PA 19104 - 4283 USA; 3 Perelman School of Medicine (PSOM) at the University of Pennsylvania, Philadelphia, PA USA; 4 Department of Neurosurgery, Center for Brain Injury and Repair, the Perelman School of Medicine (PSOM) at the University of Pennsylvania, Philadelphia, PA USA

**Keywords:** Aging-related tau astrogliopathy, ARTAG, Astrocytic plaque, Brain barrier, Hierarchical involvement, Tufted astrocyte, Ramified astrocyte, Spreading, Tau, Tauopathy

## Abstract

Aging-related tau astrogliopathy (ARTAG) describes tau pathology in astrocytes in different locations and anatomical regions. In the present study we addressed the question of whether sequential distribution patterns can be recognized for ARTAG or astroglial tau pathologies in both primary FTLD-tauopathies and non-FTLD-tauopathy cases. By evaluating 687 postmortem brains with diverse disorders we identified ARTAG in 455. We evaluated frequencies and hierarchical clustering of anatomical involvement and used conditional probability and logistic regression to model the sequential distribution of ARTAG and astroglial tau pathologies across different brain regions. For subpial and white matter ARTAG we recognize three and two patterns, respectively, each with three stages initiated or ending in the amygdala. Subependymal ARTAG does not show a clear sequential pattern. For grey matter (GM) ARTAG we recognize four stages including a striatal pathway of spreading towards the cortex and/or amygdala, and the brainstem, and an amygdala pathway, which precedes the involvement of the striatum and/or cortex and proceeds towards the brainstem. GM ARTAG and astrocytic plaque pathology in corticobasal degeneration follows a predominantly frontal-parietal cortical to temporal-occipital cortical, to subcortical, to brainstem pathway (four stages). GM ARTAG and tufted astrocyte pathology in progressive supranuclear palsy shows a striatum to frontal-parietal cortical to temporal to occipital, to amygdala, and to brainstem sequence (four stages). In Pick’s disease cases with astroglial tau pathology an overlapping pattern with PSP can be appreciated. We conclude that tau-astrogliopathy type-specific sequential patterns cannot be simplified as neuron-based staging systems. The proposed cytopathological and hierarchical stages provide a conceptual approach to identify the initial steps of the pathogenesis of tau pathologies in ARTAG and primary FTLD-tauopathies.

**Electronic supplementary material:**

The online version of this article (10.1186/s40478-018-0552-y) contains supplementary material, which is available to authorized users.

## Introduction

Deposition of pathologically altered proteins in astrocytes has been increasingly detected in neurodegenerative diseases (NDD) leading to the concept of protein astrogliopathies [33]. One main group of NDDs is associated with abnormal accumulation of the microtubule-associated protein tau, hence called tauopathies [28]. Primary tauopathies are discussed in the context of frontotemporal lobar degeneration (FTLD) as well [22]. Primary FTLD-tauopathies show a wide range of astroglial tau immunoreactive morphologies, such as tufted astrocytes in progressive supranuclear palsy (PSP), astrocytic plaques in corticobasal degeneration (CBD), ramified astrocytes in Pick’s disease (PiD) or globular astroglial inclusions in globular glial tauopathies (GGT) [22, 28]. Further tauopathies comprise argyrophilic grain disease (AGD) and neurofibrillary tangle (NFT) only dementia, which is included in the concept of primary age-related tauopathy (PART) [13, 28]. Recently, the importance of further astroglial tau pathologies such as thorn-shaped astrocytes (TSA) and granular/fuzzy astrocytes (GFA) has been emphasized and the term, aging-related tau astrogliopathy (ARTAG) has been defined [31]. In ARTAG these morphologies appear in subependymal, subpial, perivascular, white matter (WM), and grey matter (GM) locations. In a recent study, we suggested a conceptual link between GM ARTAG and astroglial tau pathologies seen in primary FTLD-tauopathies [35]. In particular, GFA-like morphologies seen in primary FTLD-tauopathies can represent an early maturation phase of astroglial tau pathology [35], analogously to the concept of pretangles and neurofibrillary tangles [3]. These aspects support the notion that astrocytes might have an early role in primary FTLD-tauopathies as recognized also for example in CBD [40]. In addition, the distribution patterns and morphology of ARTAG shows overlap with chronic traumatic encephalopathy (CTE) [35, 41, 44].

The concept of cell-to-cell propagation of neurodegeneration-related proteins was a significant step in our understanding of NDDs [18]. In the human brain this is supported by the hierarchical or stereotypical involvement of anatomical regions, defined as stages or phases [9]. However, these focus only on neuronal (tau, α-synuclein, TDP-43) or extracellular (Aβ) protein pathologies. Hence, protein astrogliopathies have not been considered in these approaches. Here we report distribution patterns of ARTAG and primary-FTLD-tauopathy related astroglial tau pathologies and address the question whether sequential stages can be recognized.

## Material and methods

### Case cohort

This study includes 687 individuals from the collection of brains from longitudinally followed subjects in the Center Neurodegenerative Disease Research (CNDR) Brain Bank at the University of Pennsylvania, Philadelphia, PA (Table 1) [2, 55]. Cases were grouped as followed: CBD (*n* = 40), PSP (*n* = 97), PiD (*n* = 22), only PART (*n* = 70), only Alzheimer disease (AD) pathology (*n* = 243), and other disorders (*n* = 215) including multiple system atrophy (*n* = 34), Lewy body disorders (*n* = 120), and amyotrophic lateral sclerosis (*n* = 61). This pooled cohort of AD plus PART plus other disorders (*n* = 528) is termed here as non-FTLD-tauopathy group. Thus for better overview we did not include PART or the presence of AGD in the group of primary FTLD-tauopathies.

**Table 1:** Demographic data of cases examined in this study

	n		Mean age		Age range		Men		Women	
Disease group	All	ARTAG	All	ARTAG	All	ARTAG	All	ARTAG	All	ARTAG
CBD	41	40	66.7	66.7	44–86	44–86	16	16	25	24
PSP	97	96	75.6	75.7	48–96	48–96	62	62	35	34
PID	22	17	67.1	66.7	31–84	31–84	13	11	9	6
PART	70	41	75.4	79.2	55–101	57–101	33	20	37	21
AD	243	152	80.5	82.3	60–98	60–98	107	71	136	81
PART/AD/Other	528	302	77.3	80.1	53–101	55–101	289	173	239	129

### Immunohistochemistry and evaluation of tau pathologies

Formalin fixed, paraffin-embedded tissue blocks from the investigated cases were evaluated. Immunostaining for tau was performed with anti-tau PHF-1 (Ser396/Ser404, 1:2000; Gift of Peter Davies). The Vectashield ABC detection kit, peroxidase/DAB, rabbit/mouse/rat (BA1000/BA2000/BA4001, 1:1000; Vector Laboratories) was used for the visualization of antibody reaction.

Following the consensus recommendations [31] we evaluated the presence (yes/no) of grey and white matter, subependymal and subpial ARTAG types in the 1) hippocampus pyramidal layers, dentate gyrus, inferior temporal gyrus, and amygdala: together representing medial temporal lobe (MTL) structures; 2) the middle frontal gyrus, anterior cingulate, inferior parietal gyrus, superior temporal gyrus and occipital cortex: together representing lobar structures; 3) the caudate-putamen, globus pallidus, thalamus, and basal forebrain: together representing subcortical structures; 4) the midbrain tegmentum, substantia nigra, locus coeruleus, pontine base, tegmentum and base of the medulla oblongata: together representing brainstem structures. Subependymal (inferior, anterior and posterior horns of the lateral ventricle, 3rd ventricle and aqueduct at different brainstem levels) locations were also evaluated. In all regions, we evaluated primary FTLD-tauopathy-related astroglial tau pathology as well. In addition, we documented the presence of neuronal and oligodendroglial tau pathology and grains.

For the subpial and WM ARTAG we stratified the regions into three groups: brainstem, basal brain regions (combination of basal forebrain and amygdala region) and lobar. For lobar regions we performed a separate analysis for frontal, temporal, parietal and occipital lobes. For subependymal ARTAG we distinguished three regions: brainstem (aqueduct), 3rd ventricle and midline of the lateral ventricle together, and the inferior horn of the lateral ventricle. For GM ARTAG and primary FTLD-tauopathy related astroglial tau pathologies, we first stratified as brainstem, subcortical (basal ganglia), MTL and lobar. Next, we performed a detailed comparison of nine regions (frontal, parietal, temporal, occipital, amygdala, striatum, substantia nigra, pons and medulla oblongata tegmentum). In the same regions we evaluated the tau pathologies using a semiquantitative score (none, mild, moderate, severe); the median of these in each region was used to generate heatmaps [21, 35].

### Conceptual approach and statistical analysis

Our approach contains 5 steps: 1) We select cases showing a specific type of ARTAG; then 2) describe patterns of involvements of different anatomical regions; followed by 3) comparison of different anatomical regions to calculate conditional probabilities, which region might precede another one; complemented by 4) logistic regression to correct for influencing factors; and finally 5) evaluating clusters of cases showing different patterns of ARTAG involvement.

We applied conditional probability analysis as reported recently for the staging of TDP-43 pathology in AD [27]. Accordingly, we compared two regions in all combinations for discordance (one affected while the other not and vice versa). The null hypothesis was that region *A* being positive while region *B* being negative and the region *A* being negative and region *B* being positive is equally likely, thus *A* and *B* region is affected at the same time (i.e., being in the same stage). McNemar’s test was used to assess the evidence against the null hypothesis. We used *p* < 0.01 to determine whether we can reject the null hypothesis. We generated a matrix for different ARTAG types involving various anatomical regions where each cell in the matrix corresponds to a conditional probability that one region is involved before another one. Conditional probability was calculated using crosstab function of SPSS.

If the conditional probability for one region is significantly higher than for the other region then we interpret that that this region is most likely affected before the other. However, this is interpreted analogously to the measurement of observer agreement for categorical data [36, 38]. Binary logistic regression models were additionally used to generate odds ratios (OR) and 95% confidence intervals (CI), where the presence of each ARTAG types in specific anatomical regions were the dependent variables, and age, sex and, as additional test, Braak stage of neurofibrillary degeneration, shown to influence the presence of ARTAG [35] were the independent variables. In case the OR > 1 with a significant *p* value we interpret this as high likelihood that two regions are affected together. In case OR < 1 with a significant p value we interpret this as low likelihood that the two regions are affected together, eventually meaning that they are affected independently from each other. For details on the methodological approach see Additional file 1.

We performed the statistical evaluation in six diagnostic groups (see case cohort). We report results for primary FTLD-tauopathies (PSP, CBD, Pick disease) and, in case there were no differences, the pooled cases of non-FTLD-tauopathies (where we included PART, AD and other disorders). We mention AD or PART cases separately only if a particular pattern is seen. We applied a significance level of 0.01 for McNemar’s test and 0.05 for logistic regression with multiple independent variables. We chose a lower significance level than the traditional 0.05 for McNemar’s test in order to reduce the likelihood of false positive findings. We provide the detailed tables of conditional probabilities and OR in the Additional file 2, Additional file 3 and Additional file 4 and here provide the combined interpretation and proposed sequential models.

In addition, we performed a hierarchical cluster analysis using the nearest neighbour approach to evaluate how cases within disease groups cluster together based on the 1) the patterns of primary FTLD-tauopathy related astroglial tau pathologies; 2) GM ARTAG with astroglial tau pathologies; and 3) subpial, WM and GM ARTAG in three major regions (lobar, subcortical/amygdala and brainstem. SPSS Statistics Version 24 was used for statistical analysis.

## Results

### Demographic summary of cases

Demographic data are summarized in Table 1. In sum 455 showed some type of ARTAG (Table 2). GM (68%) and subpial (58%) ARTAG were the most frequent, WM ARTAG was seen in 55% and subependymal in 22%. ARTAG was highly frequent in primary FTLD-tauopathies.

**Table 2:** Distribution of ARTAG types in different disease groups

		ARTAG		SP		SE		WM		GM	
Disease		n	%	n	%	n	%	n	%	n	%
All cases	no	232	33.8	192	42	357	78	203	45	145	32
	yes	455	66.2	263	58	98	22	252	55	310	68
	Sum	687	100.0	455	100.0	455	100.0	455	100.0	455	100.0
PART	no	29	41.4	16	39.0	32	78.0	25	61.0	10	24.4
	yes	41	58.6	25	61.0	9	22.0	16	39.0	31	75.6
	Sum	70	100.0	41	100.0	41	100.0	41	100.0	41	100.0
AD	no	91	37.4	59	38.8	119	78.3	39	25.7	88	57.9
	yes	152	62.6	93	61.2	33	21.7	113	74.3	64	42.1
	Sum	243	100.0	152	100.0	152	100.0	152	100.0	152	100.0
AD/PART/other	no	226	42.8	123	40.7	236	78.1	115	38.1	140	46.4
	yes	302	57.2	179	59.3	66	21.9	187	61.9	162	53.6
	Sum	528	100.0	302	100.0	302	100.0	302	100.0	302	100.0
CBD	no	0	0.0	7	17.5	29	72.5	19	47.5	1	2.5
	yes	40	100.0	33	82.5	11	27.5	21	52.5	39	97.5
	Sum	40	100.0	40	100.0	40	100.0	40	100.0	40	100.0
PSP	no	1	1.0	48	50.0	76	79.2	54	56.3	2	2.1
	yes	96	99.0	48	50.0	20	20.8	42	43.8	94	97.9
	Sum	97	100.0	96	100.0	96	100.0	96	100.0	96	100.0
PiD	no	5	22.7	14	82.4	16	94.1	15	88.2	2	11.8
	yes	17	77.3	3	17.6	1	5.9	2	11.8	15	88.2
	Sum	22	100.0	17	100.0	17	100.0	17	100.0	17	100.0

*Abbreviations*: *SP* subpial, *SE* subependymal, *WM* white matter, *GM* grey matter

### Morphology of astrocytic tau immunoreactivity

TSAs are seen in subpial, subependymal, perivascular and WM locations in all disease groups. In CBD, in subpial and perivascular location the end-feet astroglial processes are densely stained with a stubby appearance, while the cell body less (Additional file 1: Figure S1a and b); this is reminiscent of the lack of the cell body staining in astrocytic plaques. The morphology of tau immunoreactive astrocytes varies in the WM in PSP and CBD and do not always show typical TSA morphology. In the GM TSAs are less frequently seen. When present, their distribution shows a close relation to adjacent WM, such as seen in deep cortical layers, amygdala, hippocampus dentate gyrus, nucleus accumbens bordering the frontobasal WM, or in the inferior olives. This looks like as TSAs creep into the GM from the WM (Additional file 1: Figure S1c and d). Similarly, in PSP (Additional file 1: Figure S1e-g) and CBD (Additional file 1: Figure S1 h-k) cases where neuronal tau pathology is mild or lacking the astrocytic tau pathology predominates in the deeper layers together with tau positive astrocytes in the WM.

**Fig. 1:**
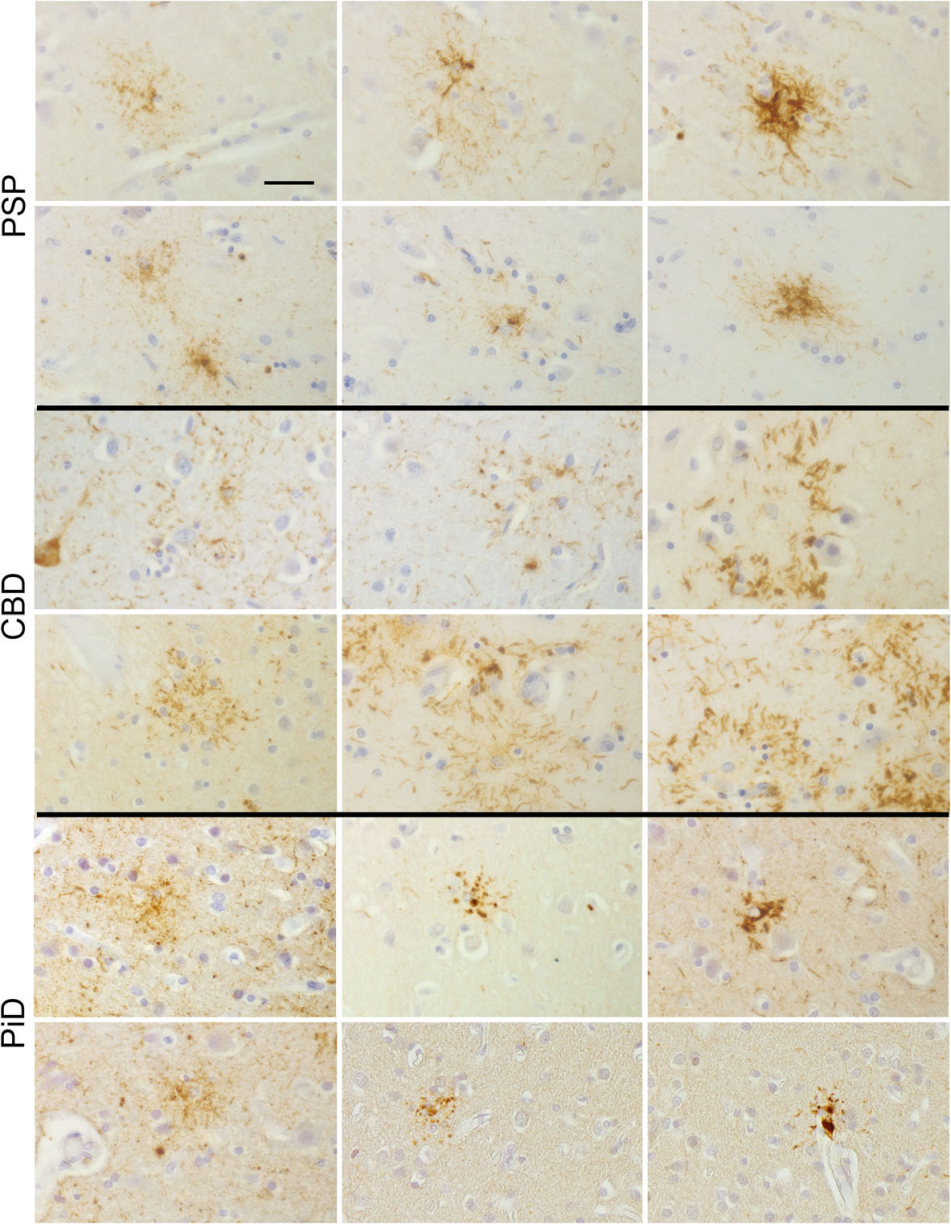
Tau immunoreactive astrocytes in progressive supranuclear palsy (PSP), corticobasal degeneration (CBD), and Pick’s disease (PiD). Note the variety of morphologies where fine granular deposits evolve into more coarse ones and then typical tufted astrocytes (PSP), astrocytic plaques (CBD), and ramified astrocytes (PiD) reminiscent of a maturation process (from left to right) of tau immunoreactive deposits. Bar represents 25 μm for all images

The predominant morphology of GM ARTAG is GFA. In cortical areas these are not specifically accumulating in the depth of the sulci. Importantly, in all primary FTLD-tauopathies various astrocytic morphologies can be recognized in the GM close to each other reminiscent of a maturation process. This includes astrocytes with fine granular tau immunoreactivity in astrocytic processes reminiscent of GFAs followed by its more coarser accumulations either in a perinuclear location or remaining in distal segments of the astrocytic arborisation (Fig. 1 and Additional file 1: Figure S2). In CBD cases astrocytic plaques clearly predominate the astroglial tau pathology in the GM. Detection of GFA-like morphology requires meticulous search due to the dense neuronal, thread and astrocytic plaque pathology (Additional file 1: Figure S3). GFA-like morphology is better seen in PSP cases together with tufted astrocytes. In PiD the GM is predominated by ramified astrocytes or by GFA-like, and additional globular–like astroglial tau immunoreacitvities. The latter two morphologies are seen particularly in areas with less or lack of neuronal tau pathology (e.g. in the occipital cortex).

### Spatial features of subpial ARTAG

**Fig. 2:**
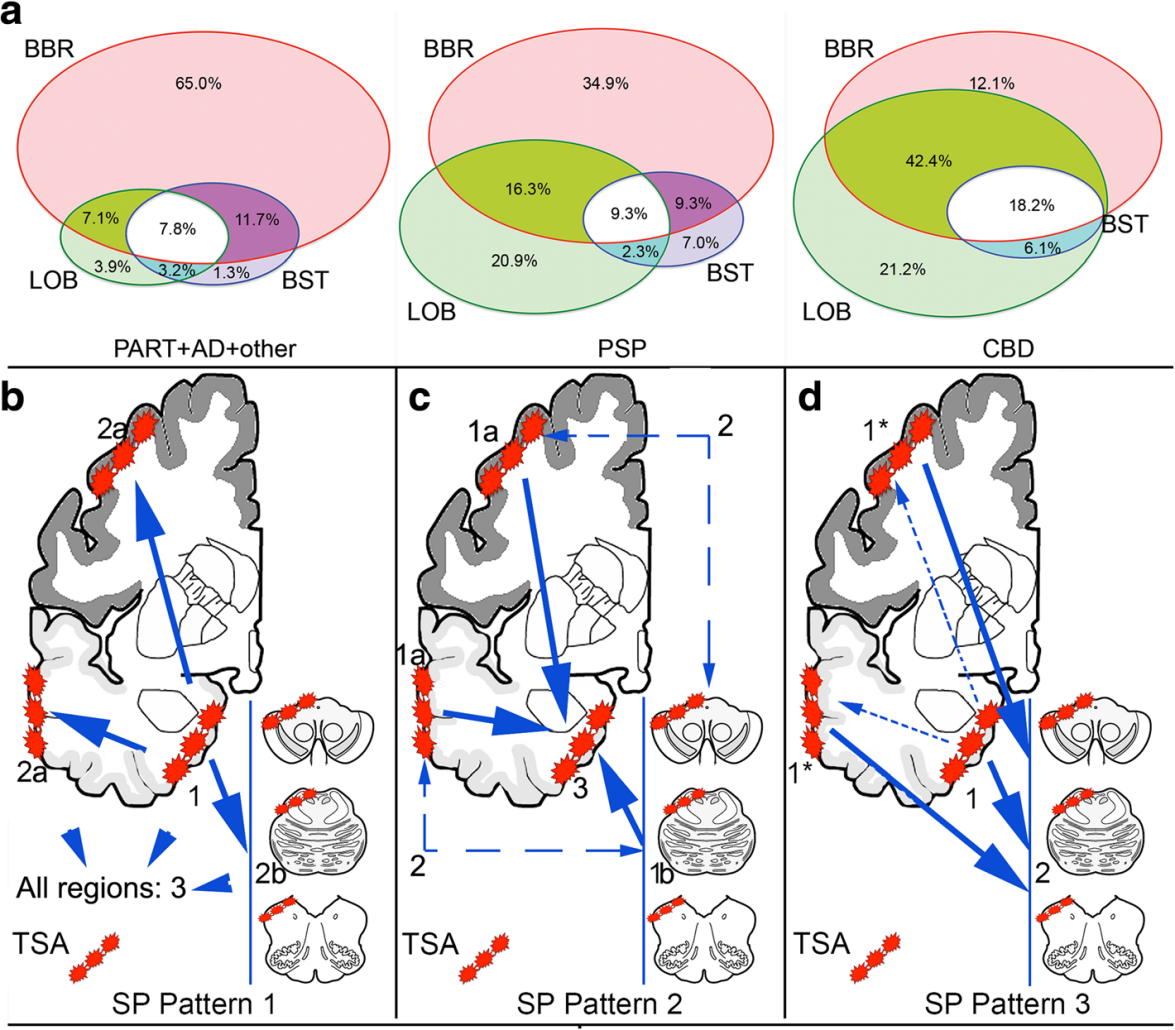
Frequencies and distribution patterns of subpial ARTAG. Frequency of subpial ARTAG in different regions (basal brain regions, BBR; lobar regions, LOB; and brainstem regions, BST) in a pooled cohort of non-FTLD-tauopathies (PART+AD+other), PSP, and CBD (**a**). Note the differences in concomitant involvement of regions. The sequential stages of subpial (SP) ARTAG in the pooled cohort of non-FTLD-tauopathies comprise pattern 1 (**b**) when basal brain regions show subpial ARTAG first (*stage 1*) followed by a bidirectional sequence rostrally (lobar) and caudally (brainstem), which two are affected rarely separately (*stages 2a or b*) and more frequently together (*stage 3*); pattern 2 (**c**) when subpial ARTAG in lobar regions or in brainstem appear first (*stage 1a or b;* two-headed dashed arrows indicate that we do not know which precedes the other); when affected together is *stage 2* and finally when additionally basal brain regions are involved is *stage 3*; and pattern 3 (**d**) as exemplified by CBD, where subpial tau immunoreactivity of astrocytic feet is the predominant pathology independently of subpial ARTAG in basal brain regions (together representing *stage 1*) and both are followed by the involvement of the brainstem, representing *stage 2*. The pathogenesis of subpial astrocyte feet tau immunoreactivity in CBD is most likely different from subpial lobar ARTAG (thus indicated with an asterisk), therefore this sequence could be termed as “masked” bidirectional. This means that the typical subpial TSAs in CBD follow the subpial ARTAG in the basal brain regions (indicated by dashed arrows) are masked by the predominant end-feet tau immunoreactivity

First we evaluated the frequency and constellations of subpial ARTAG in three major regions in cases showing subpial ARTAG: basal brain areas (amygdala and basal forebrain together), lobar and brainstem (any location for both) in different case-cohorts. In cases showing subpial ARTAG the highest frequency was observed in basal brain areas except for CBD and PiD (data not shown) where the lobar location was more frequent (Fig. 2a and Additional file 2: Table S1).

In the pooled cohort of non-FTLD-tauopathy cases, basal brain regions frequently showed ARTAG alone followed by combinations either with lobar or brainstem ARTAG or both (Fig. 2a). A few cases only showed either pure lobar or pure brainstem subpial ARTAG. Conditional probability with McNemar’s test supported the concept that subpial ARTAG in basal brain regions precedes either lobar or brainstem ARTAG. However, there are several cases where lobar and brainstem regions precede basal brain regions, in particular in PART (Additional file 2: Table S1). Neither lobar or brainstem precedes the other, rather they are present with high likelihood together. Of note, in CBD the involvement of the brainstem always follow the presence of subpial ARTAG in lobar or basal brain regions (Additional file 2: Table S1). The number of cases with subpial ARTAG in the PiD group is too low to draw conclusions.

**Fig. 3:**
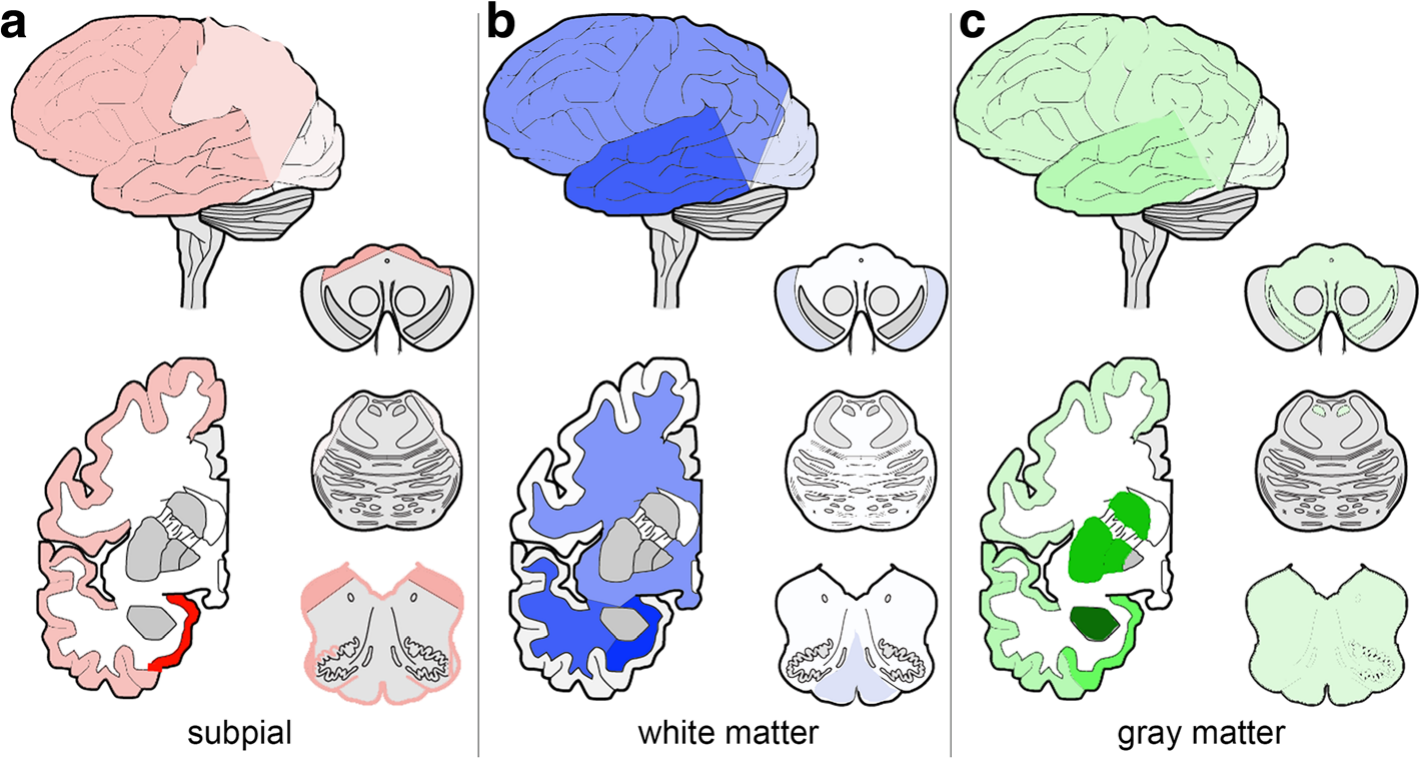
Heatmap of severity scores of subpial (**a**), white matter (**b**) and grey matter (**c**) ARTAG in the cohort of non-FTLD tauopathies. The more dark colours reflect higher severity scores

In summary, for subpial ARTAG, three patterns can be recognized. The first (Fig. 2b) is exemplified by the fact that basal brain regions show subpial ARTAG (*stage 1*). This is followed by a bidirectional sequence rostrally (lobar, *stage 2a*) or caudally (brainstem, *stage 2b*), which two, however, are usually affected together (*stage 3*). A second pattern (Fig. 2c) is when subpial ARTAG is only in lobar regions (*stage 1a*) or in brainstem (*stage 1b*) or appear together (*stage 2*) and precede that in basal brain regions (*stage 3*). These two patterns are seen in the pooled cohort of non-FTLD-tauopathies. The third pattern (Fig. 2d) is exemplified by CBD, where subpial tau immunoreactivity of astrocytic end-feet in lobar areas is the predominant pathology independently of subpial ARTAG in basal brain regions (together representing *stage 1*) and both are followed by the involvement of the brainstem, representing *stage 2*. PSP cases show overlapping features of these patterns. Heatmap of severity scores in the cohort of non-FTLD tauopathies reveals also a MTL to temporal and frontal lobe to parietal to occipital lobe and parallel also to the brainstem (Fig. 3a).

Next we were interested whether lobar subpial ARTAG shows a sequential involvement pattern (Additional file 2: Table S2). In non-FTLD-tauopathy cases there was no difference between frontal, parietal, and temporal lobes regarding which precedes the other, however, they all are affected before the occipital lobe. In CBD it seems that subpial glial end-feet tau immunoreactivity and TSAs is more likely to appear in the parietal and frontal lobes than the temporal and all precede the occipital lobe. In PSP, the frontal lobe shows significantly higher conditional probability values when compared to other lobes.

### Spatial features of subependymal ARTAG

The frequency of subependymal ARTAG was the highest in the MTL followed by subcortical and brainstem regions in all disease groups (Additional file 2: Table S3). Conditional probability analysis revealed significant results in the pooled cohort of non-FTLD-tauopathy cases and in PSP. A higher conditional probability is seen that the MTL is affected alone. Presence of subependymal ARTAG in subcortical regions seems to be independent from the MTL or brainstem. Subependymal ARTAG in the MTL usually precedes the involvement of the brainstem aqueduct. In summary, a clear sequential pattern cannot be defined.

### Spatial features of white matter ARTAG

**Fig. 4:**
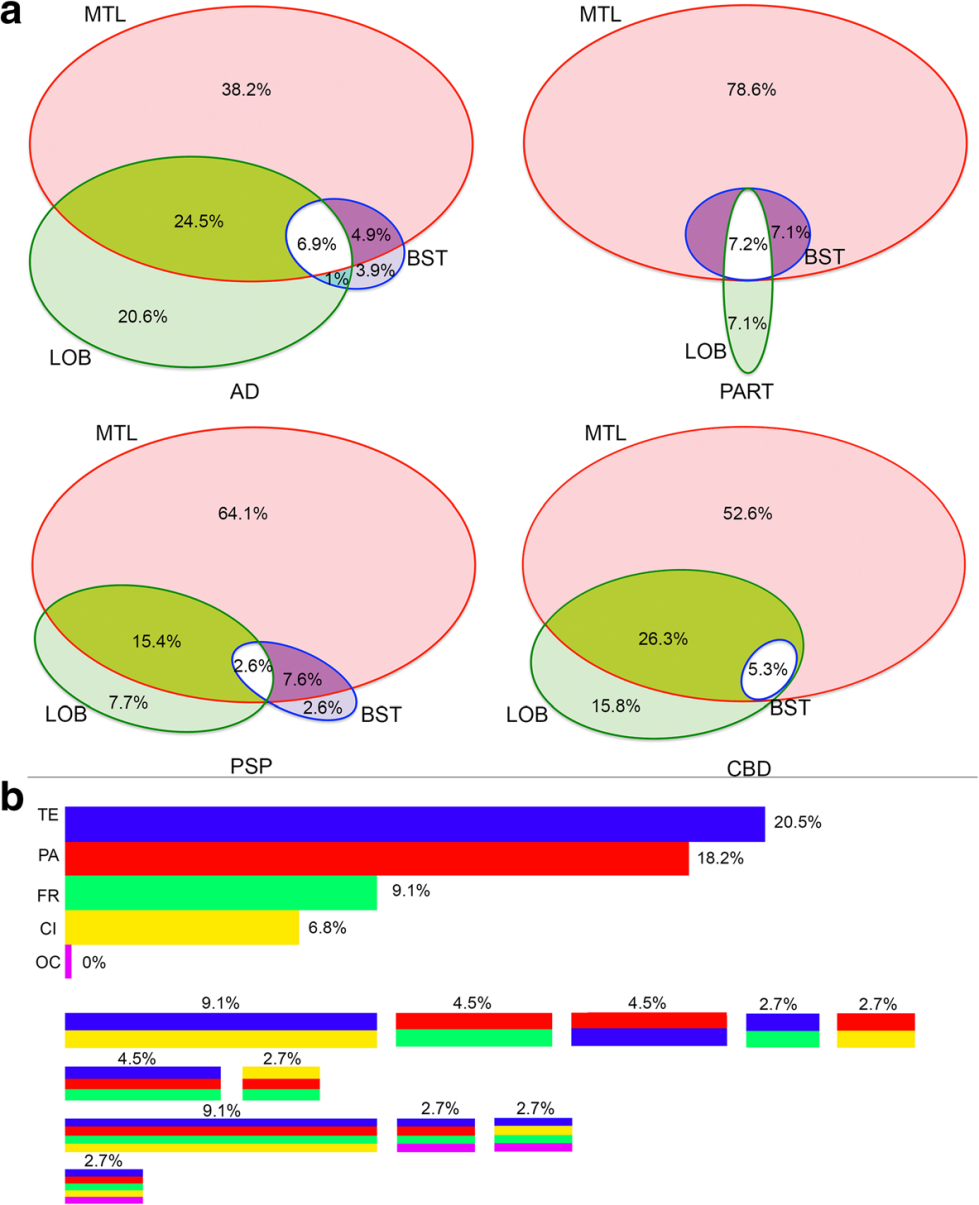
Frequency of white matter ARTAG in three major regions (**a**; medial temporal lobe, MTL; lobar regions, LOB; and brainstem regions, BST) in AD, PART, PSP, and CBD and its combinations in five lobar areas (**b**) in AD. Note the differences in concomitant involvement of regions. TE: temporal, PA: parietal, FR: frontal, CI: cingular, OC: occipital

**Fig. 5:**
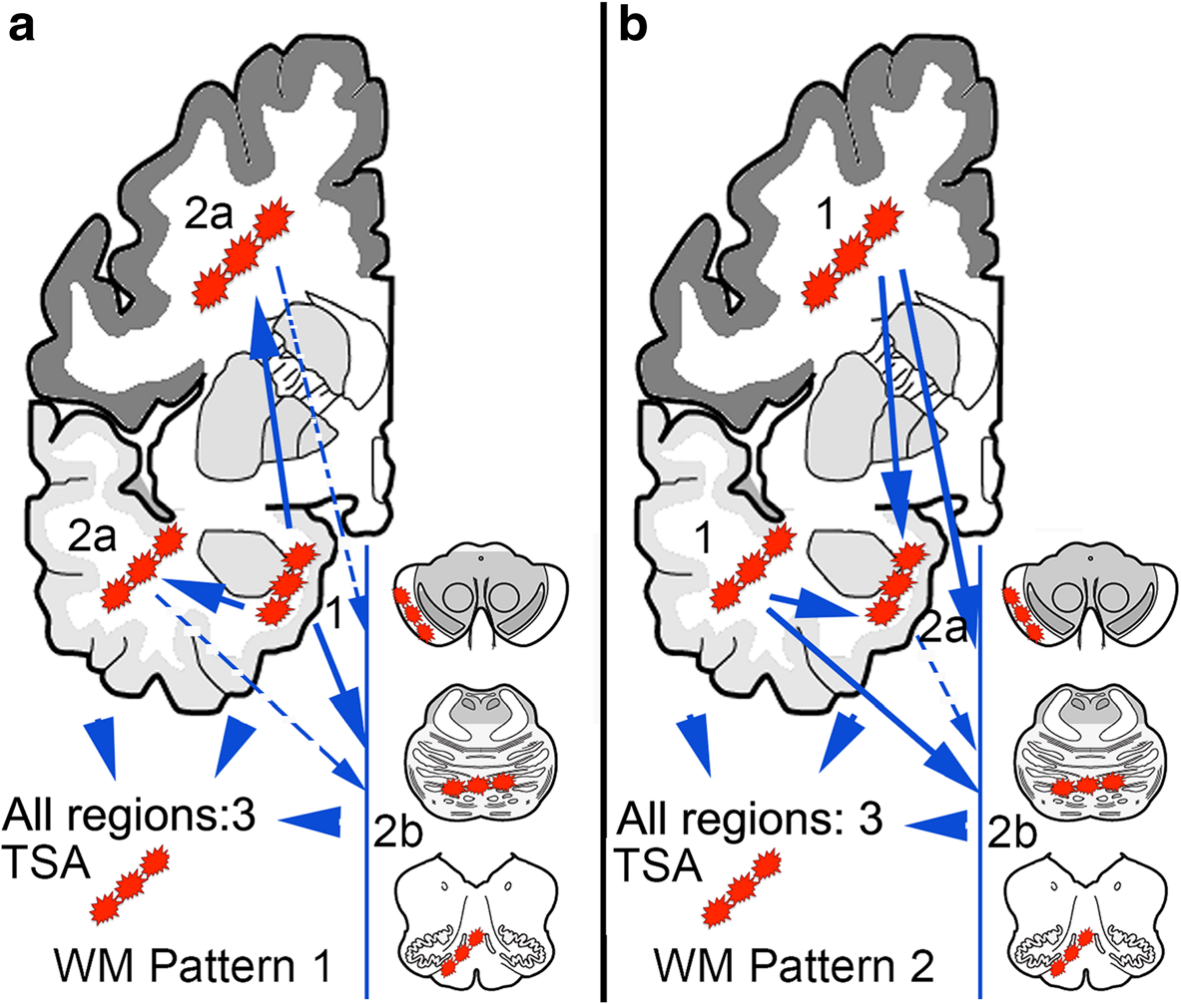
Sequential distribution patterns of white matter ARTAG in the pooled cohort of non-FTLD-tauopathies. Pattern 1 (**a**) is characterized by the appearance of white matter ARTAG in basal brain regions (*stage 1*) followed by lobar regions (*stage 2a*) or eventually brainstem (*stage 2b*) before involving all regions (*stage 3*). In Pattern 2 (**b**) lobar involvement (*stage 1*) is followed by the involvement of the basal brain regions or the brainstem (*stages 2 a or b,* respectively), before involving all regions (*stage 3*)

First we evaluated the frequency and constellations in three major regions in cases showing WM ARTAG. The highest frequency was seen in the MTL (Additional file 2: Table S4). We observe different WM ARTAG patterns for AD, CBD, PART and PSP (Fig. 4a). The pooled cohort of all non-FTLD-tauopathy cases showed combined patterns as seen for AD and PART. Interestingly, in AD cases the presence of lobar WM ARTAG without other regions involved is high (20.8%). In the pooled cohort, the MTL shows higher conditional probability values when compared to lobar regions and brainstem, and lobar higher as brainstem (fair conditional probability). Logistic regression indicates, however, that WM ARTAG in lobar regions and MTL seems to be independent from each other. This is supported by moderate conditional probability values that lobar involvement precedes the MTL. Accordingly, two patterns of sequential distribution need to be distinguished. In the majority of cases WM ARTAG appears first in basal brain regions (Pattern 1, *stage 1*) followed by lobar regions (Pattern 1, *stage 2a*) or brainstem (Pattern 1, *stage 2b*) and then all regions are involved (*Stage 3*) (Fig. 5a). However, a further pathogenesis is suggested where lobar WM seems to be independent from basal brain region. In this case lobar involvement (Pattern 2, *stage 1*) is followed by the involvement of the basal brain regions (*stage 2a*) or occasionally the brainstem (*stage 2b*) and then all regions are involved (stage 3) (Fig. 5b). Evaluating severity scores and heatmaps reveals a MTL to temporal lobe to frontal-parietal to occipital and parallel to brainstem distribution (Fig. 3b).

Next we were interested in whether lobar WM ARTAG shows a sequential involvement pattern or not. We focused only on AD cases, since these show a high frequency of lobar WM ARTAG thereby rendering this analysis feasible. Briefly, lobar WM ARTAG is usually present in frontal, parietal, or temporal lobe usually in combination of these (Fig. 4b). Thus none of these seem to precede the other, however, in any constellation of frontal/parietal/temporal WM ARTAG this precedes the presence in the occipital lobe (Additional file 2: Table S5).

### Spatial features of grey matter ARTAG

**Fig. 6:**
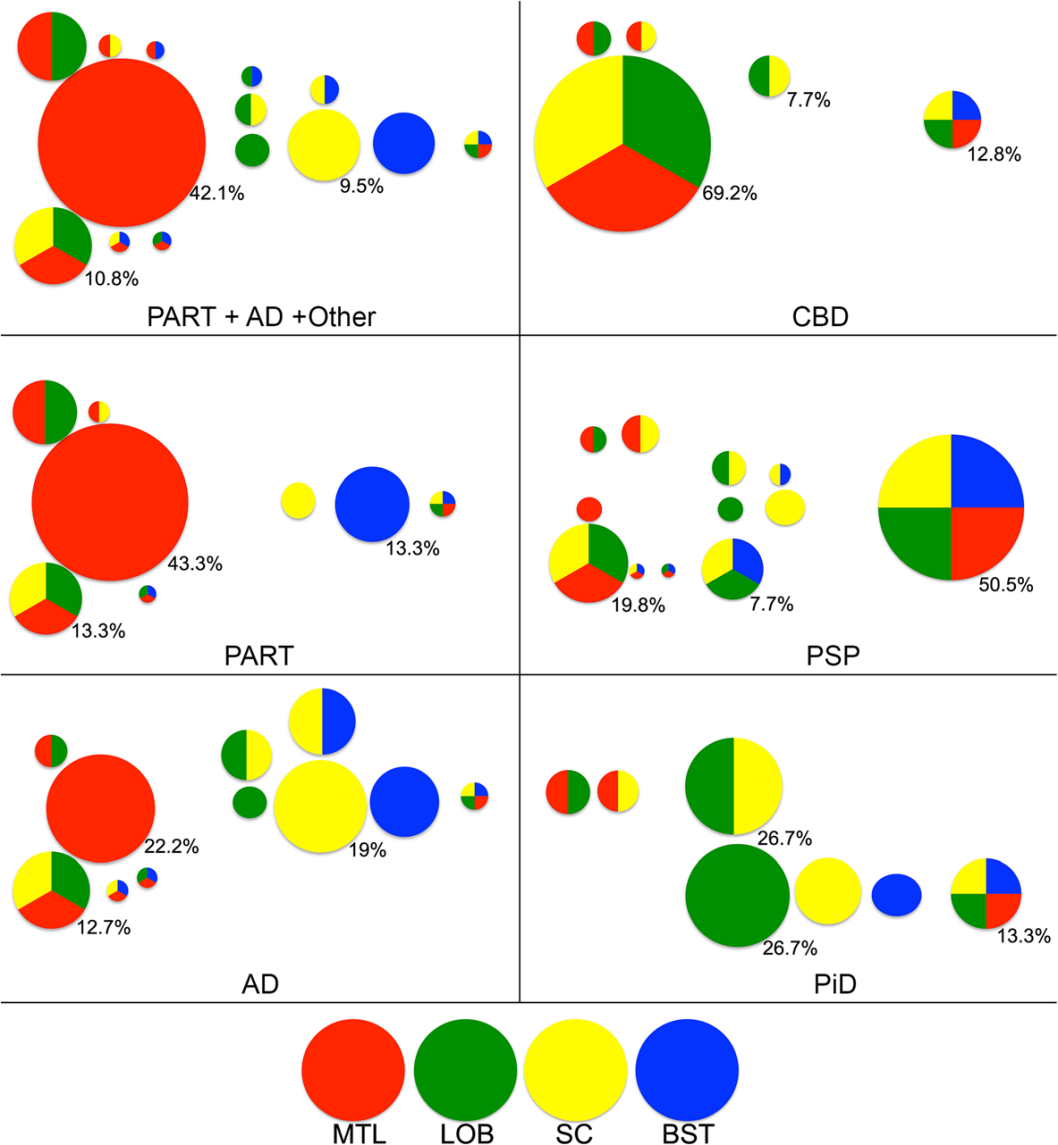
Frequency of combinations of grey matter ARTAG in different regions (medial temporal lobe, MTL; lobar regions, LOB; subcortical, SC; and brainstem regions, BST) in the pooled cohort of PART, AD and other non-FTLD tauopathies, AD, PART, PSP, Pick disease, and CBD. Note the differences and overlaps in concomitant involvement of regions. Only the three highest percentage values are shown in lower right corners for better overview. The size of the bubbles represent their frequency

First we evaluated the frequency and constellations of GM ARTAG in four major regions: MTL, lobar (pooled of frontal, parietal, temporal, and occipital lobes), subcortical (basal ganglia) and brainstem (any location) in different case-cohorts. We observed different patterns (Fig. 6 and Additional file 2: Table S6).

Non-FTLD-tauopathy cases are characterized by the predominant involvement of the MTL. A subset of cases shows pure involvement of subcortical areas or combined involvement of regions as seen in primary FTLD-tauopathies (Fig. 6). In comparisons, the MTL shows higher conditional probability then subcortical and brainstem regions then vice versa (Additional file 2: Table S6). The latter two shows fair (lobar) and moderate (brainstem) conditional probability values when compared to the MTL. Logistic regression reveals that these two regions show low ORs when compared to the MTL involvement. This suggests that there are situations when these are not affected together with the MTL. Subcortical seems to precede the involvement of the brainstem but not lobar areas reflected by a fair conditional probabilities. Higher ORs suggest that these regions are usually affected together (Additional file 2: Table S6).

To fine-tune the interpretation we evaluated the frontal, parietal, temporal, and occipital cortical regions, amygdala, striatum, substantia nigra, pons, and medulla oblongata for GM ARTAG (Additional file 3: Table S1). In the cohort of all non-FTLD-tauopathy cases the amygdala and striatum precede with fair (0.21–0.40) conditional probability cortical regions and brainstem regions. In the separated group of AD cases, for the comparisons with the striatum the conditional probabilities are in the moderate range (0.51–0.60). This is supported by higher frequency of involvement of the striatum in AD cases with GM ARTAG. The frontal, parietal and temporal cortical areas precede the occipital but with low (poor) conditional probability value. Comparison of cortical and brainstem regions show low conditional probability values but these are higher for the cortical regions. The amygdala shows fair conditional probability values when compared to brainstem areas. Logistic regression corrected for age, gender and Braak stages of neurofibrillary degenerations for this cohort of non-FTLD-tauopathy cases reveals that cortical areas are affected together with high (> 10) ORs. Importantly, high ORs are seen in the comparison of cortical areas and striatum but not with the amygdala. Brainstem areas are affected when amygdala already shows GM ARTAG (high ORs) and variably when the striatum (ORs: 0.85–1.12) is affected. Brainstem areas show high ORs when compared to each other (Additional file 3: Table S2). Finally, evaluating severity scores and heatmaps reveals a MTL and striatum to temporal lobe to frontal-parietal to occipital and parallel to brainstem distribution (Fig. 3c).

**Fig. 7:**
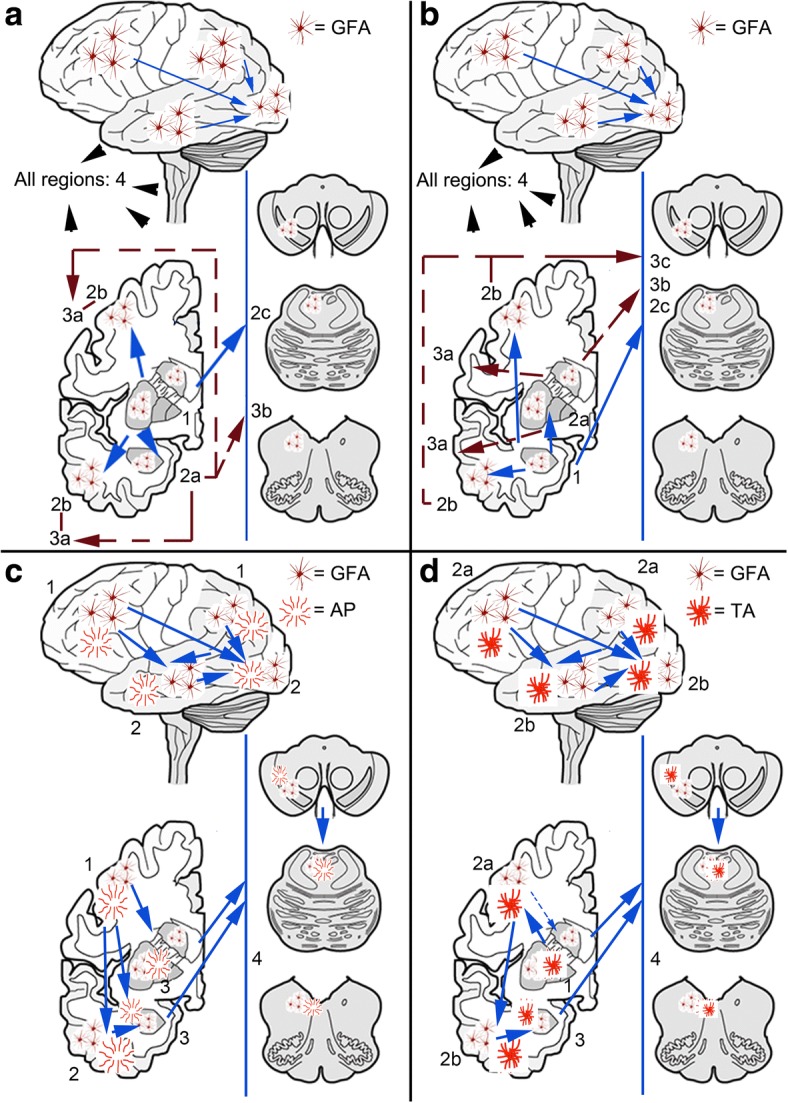
Sequential distribution patterns of astroglial tau pathology in the grey matter. In non-FTLD-tauopathies Patterns 1 and 2 are recognized. The distribution of grey matter ARTAG (granular-fuzzy astrocytes, GFA) shows Pattern 1 (**a**) characterized by the early involvement of the striatum (*stage 1*) followed by the amygdala (*stage 2a*), or cortex (here occipital is the latest to be involved) (*stage 2b*), or the brainstem (*stage 2c*); then a further region (striatum + amygdala + cortex, *stage 3a*; or striatum + amygdala + brainstem, *stage 3b*) followed by the involvement of all regions (*stage 4*). In pattern 2 (**b**) the amygdala (*stage 1*) precedes the involvement of the striatum (*stage 2a*) or the cortex (*stage 2b*), or the brainstem (*stage 2c*); then a further region (striatum + amygdala + cortex, *stage 3a*; or striatum + amygdala + brainstem, *stage 3b*; or amygdala + cortex + brainstem, *stage 3c*) followed by the involvement of all regions (*stage 4*). In CBD (**c**) the distribution of astrocytic plaques (AP) and grey matter ARTAG begins in the frontal (including premotor) and parietal cortex (*stage 1*) followed by temporal and occipital cortex (*stage 2*), paralelly moving into subcortical areas including either or both the striatum and the amygdala (*stage 3*) followed by the brainstem (*stage 4*) including the substantia nigra followed by pons and medulla oblongata. Regarding tufted astrocytes (TA) and grey matter ARTAG in PSP (**d**), a striatum (*stage 1*) to cortical (frontal-parietal to temporal to occipital) areas (*stage 2 and b, respectively*) to amygdala (*stage 3*) and to brainstem (*stage 4*), including the substantia nigra followed by pons and medulla oblongata, sequence can be recognized

Based on these findings, a dualistic model with the following sequential stages can be proposed: GFAs first appear either in the striatum or in the amygdala (Pattern 1 or 2, Stage 1). The striatal pathway (Pattern 1, *stage 1*) proceeds either towards the amygdala (*stage 2a*), cortex (*stage 2b*), or rarely to brainstem (*stage 2c*) followed by *stage 3a* (striatum + amygdala + cortex) or *stage 3b* (striatum + amygdala + brainstem) and eventually involving all regions (*stage 4*) (Fig. 7a). Note that there is no *stage 3c* since the constellation of striatum+ cortex + brainstem has not been seen in this series (Fig. 6). If we exclude cases where amygdala GM ARTAG is present this sequence patterns remains very clear (Additional file 3: Table S3), however, cortical regions show significantly higher, albeit poor conditional probability values when compared to brainstem regions. In pattern 2 the amygdala (*stage 1*) precedes the involvement of the striatum (*stage 2a*), the cortex (*stage 2b*) or very rarely the brainstem (*stage 2c*). This is followed by three combinations of *stage 3* (*a*: amygdala + striatum + cortex; *b*: amygdala + striatum + brainstem; *c*: amygdala +cortex + brainstem), and eventually followed by the involvement of all regions (*stage 4*) (Fig. 7b). This pattern is seen in cases where the striatum is not involved (Additional file 3: Table S4); here the conditional probability values are not significantly higher in cortical regions when compared to brainstem regions. There are only a few cases where a few GFAs can be noted alone in the brainstem or in the cortex.

Presence of GFA-like morphologies reveals different sequences in primary tauopathies. CBD is characterized by a fronto-parietal to temporal to occipital and to amygdala and to brainstem sequence represented by substantial to high (almost perfect) conditional probability values (Additional file 3: Table S1). However, there is a striatum to amygdala and brainstem sequence, which precedes cortical areas. PSP shows similar trends but the parietal cortex is less frequently an early affected cortical area and striatum is affected mostly before cortex. In contrast, in PiD GFA-like morphology is less frequent and therefore these sequential patterns cannot be recognized so markedly.

### Spatial features of astrocytic tau immunoreactivity in primary FTLD-tauopathies

We also evaluated classical astrocytic plaques in CBD, tufted astrocytes in PSP, and ramified astrocytes in PiD, which presented overlapping patterns with GM ARTAG in the same cohorts (Additional file 3: Tables S1 and S5).

Astrocytic plaques in CBD in the frontal, parietal and temporal regions show high conditional probabilities in comparisons with other, subcortical and brainstem, regions. Occipital shows moderate conditional probability values except for the comparison with the striatum where this is zero and the striatum shows a high value (0.95). The amygdala and striatum clearly precedes brainstem regions. Comparison of the striatum and amygdala show high values for both indicating that sequential involvement cannot be clearly defined for these two regions. It is important to recognize combined sequential patterns for GFA-like morphologies and mature astroglial tau pathologies. A four-staged sequence can be proposed: frontal (including premotor) and parietal cortex (*stage 1*) is followed by temporal and occipital cortex (*stage 2*) parallel moving into subcortical areas including either or both the striatum and the amygdala (*stage 3*) followed by the brainstem (*stage 4*) including the substantia nigra followed by pons and medulla oblongata (Fig. 7c). It cannot be defined whether the striatum or the amygdala is the first to be affected by ARTAG after the cortex, although GFAs seem to be earlier in the striatum then the amygdala (conditional probability 0.96 versus 0.50) (Additional file 3: Table S1). This is supported by heatmap evaluation as reported in our recent study [35]. In summary, the astroglial tau pathology in CBD follows a cortical (frontal-parietal- to temporal-occipital) to subcortical, and to brainstem pathway.

Regarding tufted astrocytes and GM ARTAG in PSP, the striatum shows high conditional probabilities in comparisons with other regions including the amygdala. Cortical regions and amygdala shows moderate to high values in comparison with brainstem regions. Frontal and parietal shows significantly higher conditional probabilities when compared to temporal, occipital and amygdala. Between the latter three we do find significant differences for tufted astrocytes but for GFA-type morphologies cortical regions show significantly higher conditional probability values than the amygdala. Therefore, a striatum (*stage 1*) to cortical (frontal-parietal to temporal to occipital) areas (*stage 2 a and b, respectively*) to amygdala (*stage 3*) and to brainstem (*stage 4*), including the substantia nigra followed by pons and medulla oblongata, sequence can be recognized (Fig. 7d). This is supported by heatmap-analysis as reported in our recent study [35]. Of note, however, GFA-like morphologies may appear in some cases in cortical areas before the striatum indicated by moderate to substantial conditional probability values (Additional file 3: Table S1). In summary, PSP can be described predominantly as a subcortical-cortical pattern, however, an alternative pattern is to be considered, when the involvement of the cortex parallels, or eventually precedes, the striatum.

Finally, in contrast to the lack of sequential patterns for GFA-like morphologies, ramified astrocytes in PiD appear in frontal cortex before other lobar regions and the amygdala. However, the involvement of the striatum does not clearly sequentially follow the lobar regions and may be an initiating site for astroglial tau pathologies as well since it shows fair to moderate conditional probabilities in comparisons with other regions. GM ARTAG shows similar patterns but non-significant *p* values. In summary, an overlapping pattern with PSP can be suspected either initiated in the cortex or in the striatum followed then by the other one of these and by the amygdala and brainstem areas.

### Relation of tau pathological variables in different anatomical regions

**Fig. 8:**
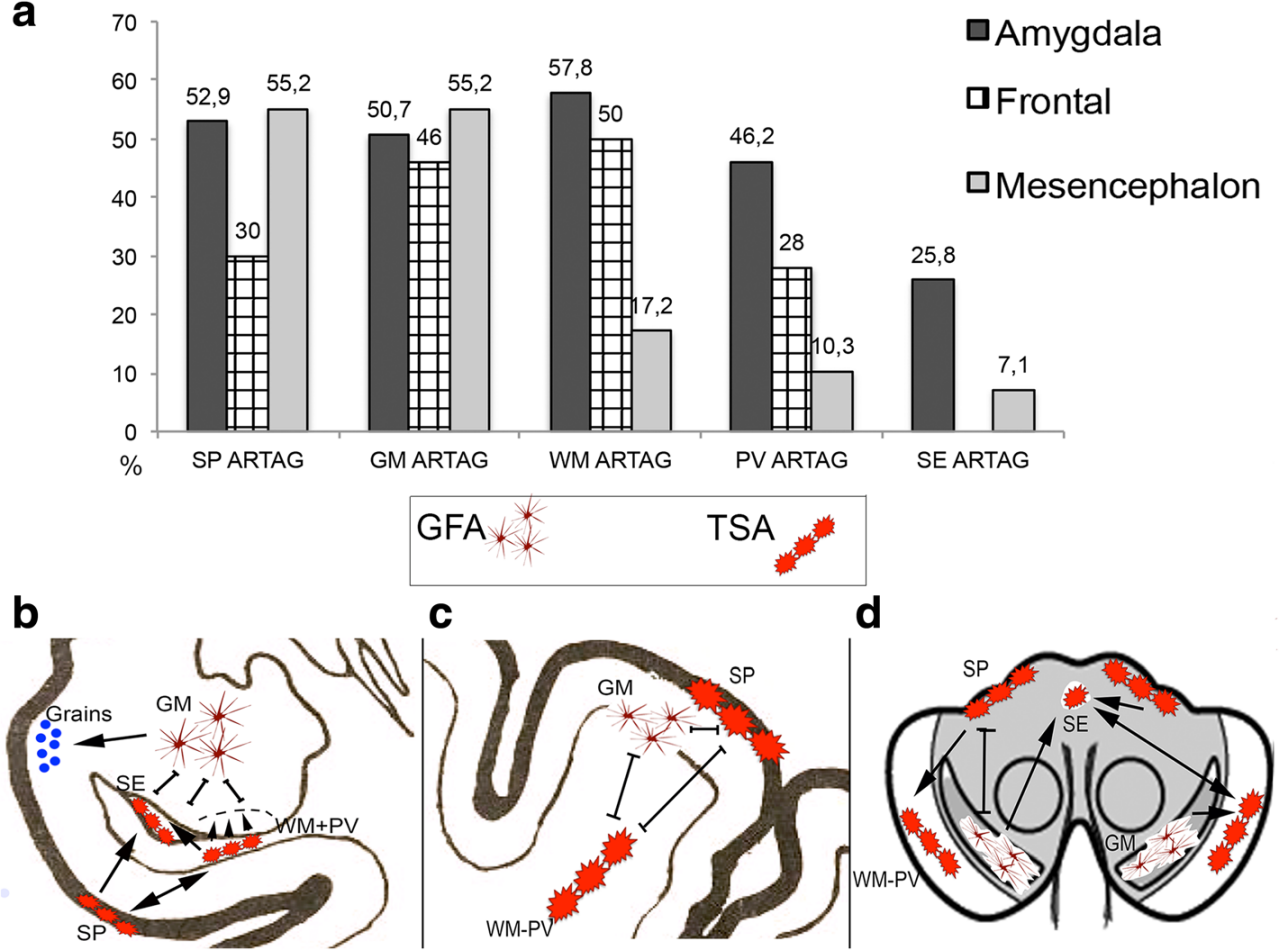
Frequency (**a**) and sequential distribution patterns of different ARTAG types in the amygdala region (**b**), frontal cortex (**c**) and mesencephalon (**d**) in pooled cases where any ARTAG type was seen. Arrows and small arrowheads indicate the proposed direction of the sequential distribution, two-headed arrows indicate concomitant, and lines the independent presence of different ARTAG types (SP: subpial, GM: grey matter, WM: white matter, PV: perivascular, SE: subependymal)

To be able to interpret the spatial features in the whole brain we need to understand whether the presence of one type of ARTAG has any effect on the appearance of a further type of ARTAG. In other words we aimed to evaluate whether one type of ARTAG precedes any other type of ARTAG in the same anatomical region. We evaluated three representative anatomical regions: amygdala as a hotspot for all ARTAG types [35], frontal cortex, and mesencephalon with substantia nigra. The frequencies of ARTAG types showed different patterns in these regions (Fig. 8a and Additional file 3: Table S6).

Briefly, in the amygdala, subpial, WM, and perivascular appear together and precede the presence of subependymal ARTAG. GM ARTAG is independent from these and shows moderate conditional probability (0.59) when compared to the presence of grains as vice versa (0.009) (Fig. 8b). This phenomenon is not seen for further comparisons of ARTAG types with the presence of grains. In the frontal area conditional probability is significant only for the comparison of WM and perivascular ARTAG (0.36 versus 0.08). Logistic regression test reveals that the presence of WM ARTAG showed significant OR with perivascular ARTAG (6.08), indicating that they are strongly associated. The OR values are significant, but lower than 1, when compared to GM (OR: 0.06), subpial (OR: 0.16) ARTAG, indicating that they are most likely independent from WM ARTAG. Subpial and GM ARTAG show fair to moderate conditional probability values when compared (Fig. 8c) and non-significant OR value (< 1). In the mesencephalon, due to the low case numbers with ARTAG the multiple regression models did not reveal significant effects; however, in univariate regression model perivascular and WM ARTAG shows significant OR (15.3) OR values. Conditional probability analysis reveals moderate to substantial conditional probability values for GM ARTAG in the substantia nigra and subpial ARTAG when compared to subependymal, WM and perivascular ARTAG. Subpial ARTAG and GM ARTAG both show high conditional probability values (0.92) in their comparison in harmony with the very low OR in logistic regression test (Fig. 8d). WM and perivascular ARTAG shows significantly high OR value.

Finally, we were interested how many cases show primary-FTLD-tauopathy related astroglial tau pathology or GFAs without any neuronal tau pathology in the same region. Interestingly, cortical areas in primary FTLD-tauopathy cases not infrequently show astroglial tau depositions (Table 3). In the pooled cohort of other disease 0.2–1.5% of all examined cases show GFAs in any region without neuronal tau pathology (Table 3).

**Table 3:** Percent of cases in different disease groups where astroglial tau pathology was seen without neuronal tau pathology

	PSP		CBD		PiD		All other	
	TA	GFA	AP	GFA	RA	GFA	TA	GFA
Frontal	1.1	5.2	2.6	2.5	0	0	0.2	1
Parietal	7.7	11	0	0	0	5	0	1.4
Temporal	4.3	3.1	2.5	2.4	0	0	0	0.6
Occipital	26.2	32.9	2.6	7.3	5.3	26.3	0	0.3
Amygdala	0	0	0	0	0	0	0	0.2
Dentate gyrus^a^	0	2.2	0	0	0	0	0	1.4
Striatum	0	0	0	0	0	0	0.4	1.5
Substantia nigra	0	0	0	0	0	4.8	0	0.6

*TA* tufted astrocyte, *AP* astrocytic plaque, *RA* ramified astrocyte, *GFA* granular/fuzzy astrocyte

^a^Note that in the dentate gyrus the morphology resembles thorn-shaped astrocytes

### Hierarchical cluster analysis

First we evaluated clustering of cases based on the presence of primary FTLD-tauopathy-related astroglial tau pathologies (i.e., tufted astrocytes, ramified astrocytes and astrocytic plaques) in three major anatomical regions (pooled lobar, subcortical and brainstem areas). Two major clusters are seen in primary tauopathies (Additional file 4: Figure S1); when all three regions or less show astrocytic tau pathology. In contrast to CBD, in PSP and PiD two further clusters are seen based on the presence of astrocytic tau pathology in two major regions (lobar and subcortical) or only in one these. In the pooled cohort on non-FTLD-tauopathies these types of astroglial tau pathologies are seen very rarely: cluster and pattern analysis and the morphology (i.e. tufted astrocytes) is reminiscent of PSP cases.

Next we added the presence of GM ARTAG in these three major anatomical regions to the cluster analysis (Additional file 4: Figure S2). In CBD and PiD this approach did not present more major clusters but more sub-clusters. In PSP GM ARTAG was usually present together with tufted astrocytes, however, there are several cases with individual constellations leading to several smaller groups. Importantly, there are PSP and PiD cases where the characteristic neuronal tau pathology is associated only with GM ARTAG (i.e., preceding immature form of astroglial tau pathologies). In the pooled cohort of non-primary FTLD-tauopathies, two major clusters are seen (presence or lack of tufted astrocytes). The majority of cases lack these astroglial tau pathologies and present small groups of cases with different constellations of GM ARTAG (see above).

Finally, we analysed cases with any type of ARTAG. Cases with GM ARTAG present a major cluster, which can be separated from those with additional WM and/or subpial ARTAG in various anatomical constellations leading to several smaller clusters consisting of a few cases (Additional file 4: Figure S3).

## Discussion

### General conceptual considerations

There are three fundamental aspects of neuropathological evaluation of neurodegenerative conditions [29]. First, clinical symptoms are thought to be associated with neuronal dysfunction and deposition of pathologically altered proteins in compartments of neurons. Second, these neurodegeneration-related proteins follow a hierarchical involvement of brain regions, which includes the likely cell-to-cell spreading of these proteins. Third, neuropathological examination of the human brain reveals changes in pathology distribution at different times in the course of a neurodegenerative disease. Together these concepts led to the development of staging strategies to describe the sequential involvement of brain regions with the aim of understanding the clinical progression [5, 7, 8, 10, 11, 21, 27, 47, 54] and defining the pre-clinical stage of diseases, which can be translated for clinical practice as in vivo biomarkers become available [14]. On the neuropathological level this means that even a few neurons showing immunoreactivity for a certain neurodegeneration-protein can be interpreted as an earliest stage of disease and not disregarded as a non-specific finding. These concepts, however, are based on a neuron-centric view of neurodegenerative diseases, in particular that stages are considered to follow neuronal networks.

The importance of astrocytes in neurodegeneration is increasingly recognized [15, 33, 57]. To evaluate astrocytes, however, a distinct conceptual approach is needed. Astrocytic networks are being recognized; furthermore, astrocytes show a complex spectrum of functions, which are associated with neurons as well as brain barriers [15, 33, 57]. The concept of protein astrogliopathies is mostly implemented for the diagnostic classification of tauopathies [33]. Astrocytic morphologies such as astrocytic plaques and tufted astrocytes should be considered as a dynamic process. Accordingly, PSP, CBD, and PiD are characterized by well-defined morphologies; however, these are the end stage of cytopathological maturation when protein pre-aggregates ripe into larger aggregates and finally form dense protein accumulations in certain segments of the astrocytes (Fig. 1). The first step of fine granular accumulation in astrocytic processes is followed by the transportation to distal or proximal segments of the astrocytic cytoskeleton and then by formation of aggregates that become argyrophilic and/or ubiquitinated [20, 33, 35, 48]. This interpretation provides a conceptual link between GFAs of ARTAG and primary FTLD-tauopathy-related astrocytic morphologies [33, 35].

Here we attempted to evaluate the patterns of ARTAG types. As expected the relative purity of neuron-based staging systems cannot be reproduced. As a limitation of our study we did not evaluate whole hemispheric sections and some of ARTAG types might have been missed in certain anatomical regions. However, analysis of patterns allows us to identify principles to appreciate better the heterogeneity of ARTAG in diverse NDD and pave the way to improved understanding of pathogenesis. First of all, our study supports the notion that the pathogenesis of GFA in the GM must be distinguished from TSAs in the subpial, subependymal, WM and perivascular locations [35]. Second, we show here that there is a regional difference in the interplay between ARTAG types represented by TSAs, e.g. subpial, WM and subependymal. This is exemplified by the observation that WM ARTAG is independent from the development of subpial ARTAG in lobar regions whereas strongly associates with that in basal brain areas and brainstem (see Fig. 8). Therefore, the interpretation of the sequential distribution of ARTAG types needs to consider further and local pathological variables. We applied several levels of evaluation to propose sequential patterns; our observations argue against an exclusive path for hierarchical involvement for any ARTAG type. This suggests distinct pathogenic events initiating ARTAG at specific locations, which then show sequential involvement of further anatomical areas.

### Sequential patterns of subpial, subependymal and white matter ARTAG

The distribution patterns of these three ARTAG types show considerable overlap. In addition, perivascular ARTAG is prominently associated with WM ARTAG and therefore not evaluated separately. Based on our observations, stages cannot be defined for subependymal ARTAG. It seems that in most cases subependymal ARTAG follows the development of subpial ARTAG suggesting that the pathogenic event, inducing subependymal ARTAG, needs to be more prominent or requires longer duration than in the situation when only subpial ARTAG develops. As discussed already, the aetiology of subpial ARTAG might be associated with dysfunction of the brain barriers [35, 41]. This is strongly supported by the prominent astroglial expression of connexin-43 and aquaporin-4 correlating with ARTAG [37]. Importantly, acute perivascular cellular uptake of blood-borne proteins is prominent in astrocytes and neurons, but not microglia in experimental concussion suggesting brain barrier disruption as a feature of concussion [25].

One pattern of subpial ARTAG seems to be initiated in basal regions proceeding towards the convexity of the brain (lobar areas) or dorsolateral parts of the brainstem. A second pattern, however, most likely begins in the convexity of the brain paralleled by the brainstem involvement but preceding the basal brain regions. Two aspects need to be considered for the interpretation: a pathogenesis related to the circulation of the CSF or local mechanical factors. During the circulation of the CSF from the lateral ventricles it enters the aqueduct and then the cerebellomedullary cistern at the brainstem level (i.e. via the foramen of Magendie and foramina of Luschka). The fluid then circulates in the subarachnoid space and reaches the basal areas before proceeding to the convexity. During the pulsatile flow of the CSF, the vascular expansion following cardiac systole occurs first at the base of the brain reversing then the flow of cisternal CSF superimposed by a circadian or diurnal rhythm [1]. Thus, subpial ARTAG might reflect the consequences of a “traffic jam” of CSF-flow at basal brain regions associated with the disturbance of CSF-brain barriers and with or without qualitative changes in the CSF. In this model the brainstem and the convexity develops ARTAG only later since the flow of the CSF may be less disrupted in these locations. A similar pattern of WM ARTAG mirrors this, in particular that in the initiating site of the amygdala, subpial and WM ARTAG strongly associates with each other. Interestingly WM tracts are important for oedema fluid movement and clearance [1]. Of particular note is the progression of WM ARTAG towards the occipital lobe from other lobar areas, which is reminiscent of the progression of NFT pathology in AD as suggested by the Braak stages [5]. Indeed, lobar WM ARTAG is frequent in AD [35], moreover, the possible role of cerebral arteries and the pulsatile CSF flow in the spreading of NFT degeneration has been also proposed based on other meticulous observations [45].

The existence of a second pattern of subpial and WM ARTAG raises the possibility of further pathogenic events such as a history of repeated mild traumatic brain injury (TBI) or a single severe TBI, where diffuse axonal injury across the WM is thought to be an important pathological feature [24]. Regarding subpial ARTAG, the early appearance of TSAs in the convexity of the brain and lateral parts of the brainstem raises the possibility of local mechanical compression. This would be analogous to the development of TSAs in the spinal cord in cervical spondylosis [50]. Subpial TSAs are frequent in CTE [44]. Blast injury has been also reported to be associated with tau positive astrocytes mainly in the frontal and parietal cortices [51]. It must be noted that in our series ARTAG was frequently not associated with the characteristic constellation of concomitant NFT pathology and ARTAG in the depth of the sulci as suggested for CTE [43]. However, our observations on two major patterns of subpial and WM ARTAG support the notion that the development of ARTAG and CTE type pathology shares common pathogenesis. In summary, it could be theorised that the basal region-to-convexity pattern is initiated by a disturbance in CSF circulation, while the convexity-to-basal brain region pattern might be initiated by, or associated with, mechanical perturbations of the brain such as occurs with mild TBI. The proposed spreading sequence of subpial and WM ARTAG could then most likely be linked to mild TBI and to alterations of the pulsatile flow of the CSF and interstitial fluid over the lifespan. As for TBI and CTE, the relative “dose”, such as the frequency and severity of injury [23], associated with these pathologies is yet unknown.

Importantly, subpial astroglial tau pathology is different in CBD, in particular in lobar areas, i.e., the convexity of the brain, where the morphology of subpial TSAs is different in CBD. These are mostly the astrocytic feet immunostained and less the cell body as in typical TSAs, although a few TSAs can be recognized as well. Lobar subpial TSA always associates with tau pathology in the GM and WM contrasting non-FTLD tauopathies where lobar subpial ARTAG can be present alone. The pathogenesis of subpial astrocyte feet tau immunoreactivity in CBD is most likely different from subpial lobar ARTAG. Therefore the sequence (i.e., involvement of the convexity and basal brain region in *stage 1*) identified in CBD could be termed as “masked” bidirectional. This means that the basal brain regions-to-convexity and bidirectional to brainstem sequence seen in non-FTLD-tauopathies with the typical subpial TSA morphologies representing typical subpial ARTAG are masked by the predominant end-feet tau immunoreactivity.

### Sequential patterns of grey matter ARTAG

Interpretation of the observations on astrocytic tau pathology requires an approach on two conceptual levels. First, at a cellular level the recognition of maturation phases of tau accumulation is important. Second, on the anatomical level, the relationship of astrocytic tau pathology to neuronal tau pathologies varies between anatomical regions. The most important finding of our study is that for GFAs in non-FTLD-tauopathies we can recognize a striatal and amygdala pathway each proceeding to cortical areas and brainstem. The striatal pattern is clearly reminiscent of the combined pattern of tufted astrocytes and GFAs seen in PSP. We are aware of the description of so called equivocal tufted astrocytes in pallido-nigro-luysian-atrophy showing different morphology and distribution [59], however, in our study we evaluated PSP cases with unequivocal tufted astrocytes. In our cohort the non-FTLD-tauopathy group included a wide range of neurodegenerative conditions, including PART cases. In PSP, as well as CBD and PiD, we have found a dissociation of the density of neuronal and astroglial tau pathologies [35]. There are also reports on the appearance of astroglial tau pathology in areas lacking neuronal tau pathology [21, 40]. Ling et al. [40] and Josephs et al. [26], by examining CBD and PSP cases, respectively, speculated that neuronal pathology is abundant in end-stage disease and therefore gradually overtake astroglial tau pathology. All together these support the notion that cases with concomitant early features of primary FTLD-tauopathies might be more frequent than previously assumed [35]. Even more, some of the cases with prominent GM ARTAG without prominent features of other disorders can be associated with clinical symptoms [34].

An interesting observation of our study is the high conditional probability that GFAs precede grains in the amygdala. Indeed GFAs are consistent finding in AGD [4, 16, 56]. We propose that cases with GFAs in the amygdala and without characteristic grains could be interpreted as pre-AGD pathologies. Interestingly, we observed this phenomenon in psychiatric conditions [35] and on the other hand psychiatric symptoms are not unusual in AGD [28]. In CBD tau pathology in the amygdala is probably mild in very early disease stages in the absence of secondary pathology [40], and eventually develops more when grain pathology, either as feature of CBD [53], or as additional AGD, is also present.

Importantly, we observed cases, which showed astroglial tau deposits with the lack of neuronal tau pathology in the same region. The question is whether astroglial tau pathology might precede neuronal tau pathology? We hypothesize that astroglia either phagocytizes pathological tau derived from the endings of projecting neurons or we observe local astroglial upregulation of tau as a response to a yet unidentified event. Alternatively, a reservoir of phosphorylated tau might be continuously released upon lysis of the axons as seen for example in TBI, where axons continue to degenerate for years after injury, a process that includes accumulations of phospho-Tau [24]. In some areas this might be again linked to CSF-brain barrier dysfunction, reflected by increased connexin-43 and aquaporin-4 expression in GM ARTAG-bearing astrocytes [37], supported also by observations that tufted astrocytes as well as astrocytic plaques tend to be positioned in close proximity to blood vessels [49]. Astrocytes do have phagocytic receptors and have been show to internalize or engulf pathological alpha-synuclein and most likely play a role in clearance and their degradation [12, 30, 39, 42, 46]. However, for tau this is not yet clearly defined. Experimental studies in tau transgenic mouse model of astrocytic tau pathologies suggest that this contributes to glial degeneration [19], and as a consequence of astrocytic tau pathology neuronal degeneration can be detected in the absence of neuronal tau inclusions [17]. Pattern analysis [35] indicates that neuronal tau is usually present locally where astroglial is seen or in projection areas. Some studies suggest dysfunction of pathological tau-harbouring protoplasmic astrocytes associated with neuronal dysfunction [48, 52]. This could help to better understand the relevance of astroglial tau pathology e.g. in the amygdala even without prominent tau pathology.

### Implications for staging astroglial tau pathologies

Clear staging systems for PSP, CBD and PiD, such as for NFT pathology in AD [5] or Lewy bodies in Parkinson disease [6], are lacking. However, there are several studies indicating sequential distribution of pathologies [21, 58]. The wide spectrum of clinical presentations and pathological heterogeneity [22, 28] associated with these disorders hamper the development of uniform staging protocols. What lessons can be learned from our study? First, that the striatum, amygdala and cortex (mostly frontal-parietal) can be an initiating site to develop astroglial tau pathology. This can be a pure finding indicating a pathogenic event in these locations or combined (i.e. secondary) to the presence of neuronal tau pathology (i.e. in the form of pretangles) in the same region or in areas projecting to these regions (i.e., substantia nigra projecting to striatum or subcortical projecting to cortex). In later stages of the disease neuronal tau pathology increases in these locations. As a further aspect some types of tauopathies characterized by different tau strains may differ in the predominance of neuronal tau pathology, exemplified by PSP cases with prominent brainstem neuronal tau pathology with relative lower density of astroglial tau in other regions or the predominance of astroglial tau pathology in unusual locations such as the hippocampus [32]. Most likely strain specific staging systems should be considered and not unifying for all CBD or PSP cases. In this respect it is extremely important to recognize cases with low amount of GM ARTAG in certain areas theoretically representing pre-stages of one of the strains of primary FTLD-tauopathies.

**Fig. 9:**
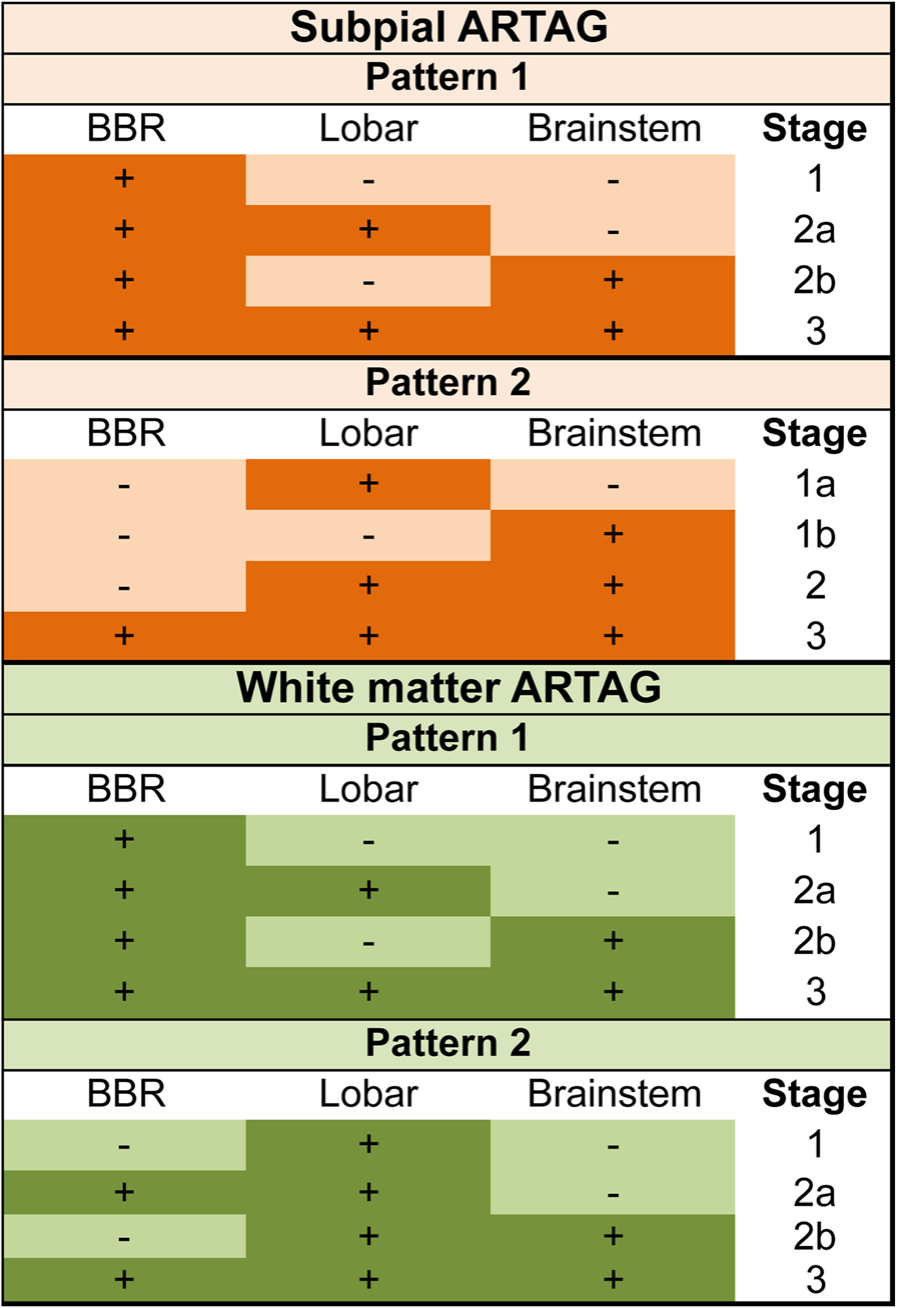
Staging scheme for subpial and white matter ARTAG

**Fig. 10:**
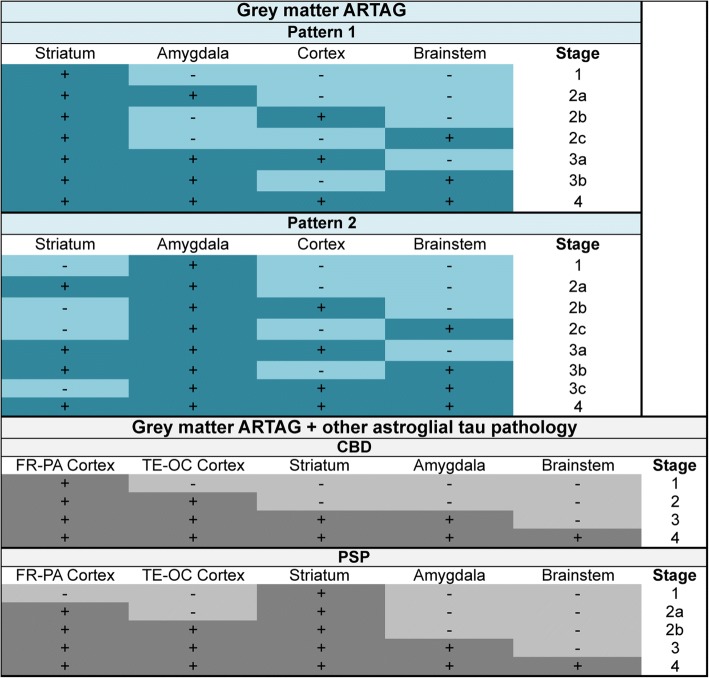
Staging scheme for grey matter ARTAG and for all astroglial tau pathologies in corticobasal degeneration (CBD) and progressive supranuclear palsy (PSP). FR: frontal, PA: parietal, TE: temporal, OC: occipital cortex

For the practising neuropathologists an approach to the staging is summarized in Figs. 9 and 10. For subpial, WM, and GM ARTAG the final stages of different patterns are similar, thus the initiating pattern cannot be defined (i.e. only the stage number). Similarly, for GM ARTAG the initiating site cannot be defined when *stages 3a* or *3b* or 4 are seen. For primary FTLD-tauopathy related tau-astrogliopathies (combination of GFAs, tufted astrocytes or astrocytic plaques) the sequential stages can be better recognized (Fig. 10).

## Conclusions

Do the observations on subpial, subependymal and WM ARTAG have urgent therapeutic consequences? This remains to be seen since ARTAG was only defined clearly very recently and there is a need for more clinicopathological studies of ARTAG. This notwithstanding, the observations reported here reflect the various impacts the human brain undergoes during life, which may have effects on the physiological functioning of brain barriers. Developing in vivo markers for these ARTAG types or dysfunction of the CSF-brain barrier will help to understand their role in the pathogenesis of neurodegenerative conditions and eventually lead to better stratification of patients for therapies. For example therapies that require effective functioning of these barriers might be less advantageous for those individuals with prominent ARTAG. For GM ARTAG we show different patterns suggestive of complex relationships with other pathological alterations and eventually spreading mechanisms of astroglial tau pathologies. It will also be important to determine if there is glial cell-to-cell spread of ARTAG or spread of tau pathology between neurons and glia. The overlap of distribution patterns of GM ARTAG in diverse disorders and astroglial tau pathologies of primary-FTLD tauopathies supports the concept of common initiating events and pathogenesis.

## Additional files

Additional file 1: Summary of the statistical method and morphology of astroglial tau pathologies. (PDF 2229 kb)

Additional file 2: Pairwise conditional probability matrix and odds ratios of different ARTAG types. (PDF 876 kb)

Additional file 3: Pairwise conditional probability matrix and odds ratios of grey matter ARTAG and primary FTLD-tauopathy associated astroglial tau immunoreactivities. (PDF 437 kb)

Additional file 4: Hierarchical cluster analysis of astroglial tau pathologies in different disorders. (PDF 1463 kb)
