# Supplementary material for: Sequential stages and distribution patterns of aging-related tau astrogliopathy (ARTAG) in the human brain
Source: Acta Neuropathol Commun. 2018 Jun 11;6:50. doi: 10.1186/s40478-018-0552-y (PMC5996526; doi:10.1186/s40478-018-0552-y)
Supplement: Supplementary file 1 — Summary of the statistical method and morphology of astroglial tau pathologies. (PDF 2229 kb) [file 40478_2018_552_MOESM1_ESM.pdf]

Sequential stages and distribution patterns of aging-related tau astrogliopathy (ARTAG) in the human brain

Gabor G. Kovacs[1,2], Sharon X. Xie[3], John L. Robinson[2], Edward B. Lee[2], Douglas H. Smith[4], Theresa Schuck[2], Virginia M.-Y. Lee[2], and John Q. Trojanowski[2]

1: Institute of Neurology, Medical University of Vienna, Vienna, Austria; 2: Center for Neurodegenerative Disease Research (CNDR), Institute on Aging and Department of Pathology & Laboratory Medicine; 3: Department of Biostatistics and Epidemiology; 4: Department of Neurosurgery, Center for Brain Injury and Repair, the Perelman School of Medicine (PSOM) at the University of Pennsylvania, Philadelphia, PA, USA.

SUPPLEMENTAL FILE 1

Summary of the statistical method

We compared two regions in all combinations for discordance (one affected while the other not and vice versa). The null hypothesis was that region *A* being positive while region *B* being negative and the region *A* being negative and region *B* being positive is equally likely, thus *A* and *B* region is affected at the same time (i.e., being in the same stage). McNemar`s test was used to assess the evidence against the null hypothesis. We used $p<0.01$ to determine whether we can reject the null hypothesis. We generated a matrix for different ARTAG types involving various anatomical regions where each cell in the matrix corresponds to a conditional probability that one region is involved before another one. Conditional probability was calculated using crosstab function of SPSS. The conditional probability of region *A* to precede region *B* or vice versa was calculated as follows:

Region A	Region B	
	ARTAG present	ARTAG not present
ARTAG present	W	X
% of cases within region A	%	%
% of cases within region B	%	*conditional probability A preceding B*
ARTAG not present	Y	Z
% of cases within region A	*conditional probability B preceding A*	%
% of cases within region B	%	%

This represents the fraction of cases where region A is affected before region B (i.e., X/(X+Z)) or that region B is affected before region A (i.e., Y/(Y+Z)). If the conditional probability for one region is significantly higher than for the other region then we interpret that that this region is most likely affected before the other. However, this is interpreted analogously to the measurement of observer agreement for categorical data [1, 2], where Kappa value is above 0.81 it is considered almost perfect, 0.61–0.80 as substantial, 0.41-0.60 as moderate, and 0.21-0.4 as fair and <0.21 as poor agreement. Accordingly, if the conditional probability is low (<0.21) for one compared region this indicates that it less likely to precede the involvement of the other region. If both compared regions are <0.21 we interpret that they are usually affected at the same time (together) since we cannot demonstrate that one is more likely before another one. If the conditional probability is >0.40 for one compared region this indicates that this regions most likely precedes the involvement of the other one. If it is higher for both (>0.40) we interpret that they are affected most likely independently from each other, and sequential progression cannot be demonstrated. Moreover, these interpretations, in particular for values between 0.21-0.60, must be in harmony with the patterns observed including the frequencies of involvement and the odds ratios (OR) in logistic regression models. Thus, binary logistic regression models were additionally used to generate odds ratios (OR) and 95% confidence intervals (CI), where the presence of each ARTAG types in specific anatomical regions were the dependent variables, and age, sex and, as additional test,

Braak stage of neurofibrillary degeneration, shown to influence the presence of ARTAG [3] were the independent variables. In case the OR >1 with a significant p value we interpret this as high likelihood that two regions are affected together. In case OR < 1 with a significant p value we interpret this as low likelihood that the two regions are affected together, eventually meaning that they are affected independently from each other.

References

1 Kovacs GG, Xie SX, Lee EB, Robinson JL, Caswell C, Irwin DJ, Toledo JB, Attems J, Bencze J, Bieniek KFet al (2017) Multisite assessment of aging-related tau astrogliopathy (ARTAG). J Neuropathol Exp Neurol 76: 605-619 Doi 10.193/jnen/nlx.041

2 Landis JR, Koch GG (1977) The measurement of observer agreement for categorical data. Biometrics 33: 159-174

3 Kovacs GG, Robinson JL, Xie SX, Lee EB, Grossman M, Wolk DA, Irwin DJ, Weintraub D, Kim CF, Schuck Tet al (2017) Evaluating the Patterns of Aging-Related Tau Astrogliopathy Unravels Novel Insights Into Brain Aging and Neurodegenerative Diseases. J Neuropathol Exp Neurol 76: 270-288 Doi 10.1093/jnen/nlx00

Figure 1. Morphology of astroglial tau immunoreactivities.

Subpial region in the occipital cortex in a representative CBD case showing stubby astroglial end-feet and less thorn-shaped morphology (**a** and enlarged in **b**). Amygdala in a case with ARTAG showing TSAs in the white matter and in the adjacent grey matter (**c** and enlarged in **d**). Occipital white matter and cortex in a representative PSP case (**e**) showing predominance of tufted astrocytes in the deeper cortical layers (enlarged in **f**) and tau-positive astrocytes in the white matter (see arrowhead in **g**). Occipital region in representative CBD cases (**h-k**) with tau positive threads and astrocytes in the white matter and in deeper cortical layers and single astrocytic plaques (see arrowhead in **j**) and lack of neuronal tau pathology (box in **h** and **j** is enlarged in **i** and **k**. respectively). Bar in image "**a**" represents 75 μm for **a**, 30 μm for **b**, 150 μm for **c**, **e**, **h**, and **j**, 100 μm for **d**, 50 μm **f**, **g**, **i** and **k**.

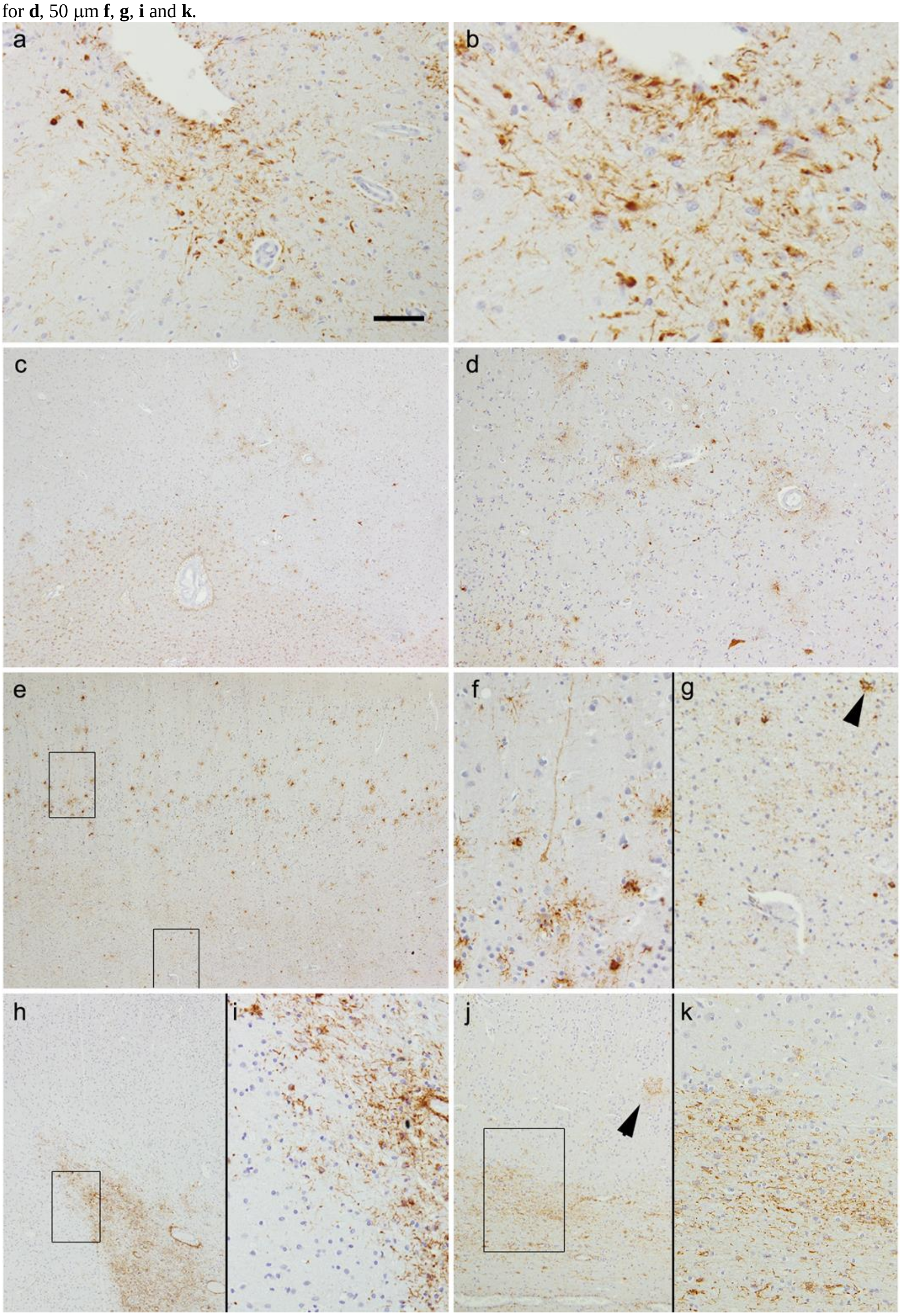

Figure 2. Tau immunoreactive astrocytes in progressive supranuclear palsy (PSP), corticobasal degeneration (CBD), and Pick`s disease (PiD). This image is a highly contrasted version of Figure 1 of the publication.

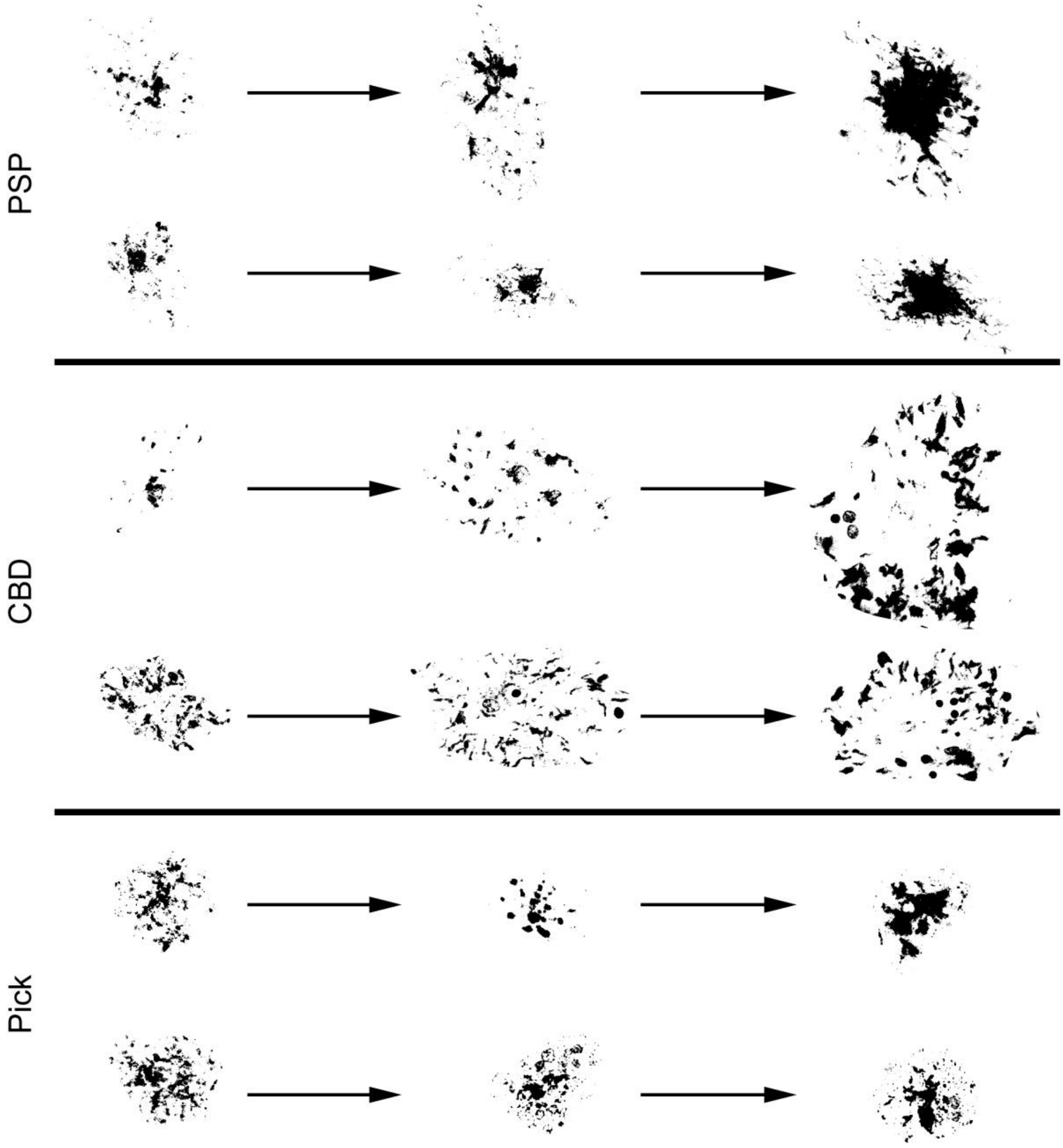

Figure 3. Examples of astroglial tau immunoreacitivities in different CBD cases representing pre-forms but not mature astrocytic plaques and in part reminiscent of granular/fuzzy astrocytes.

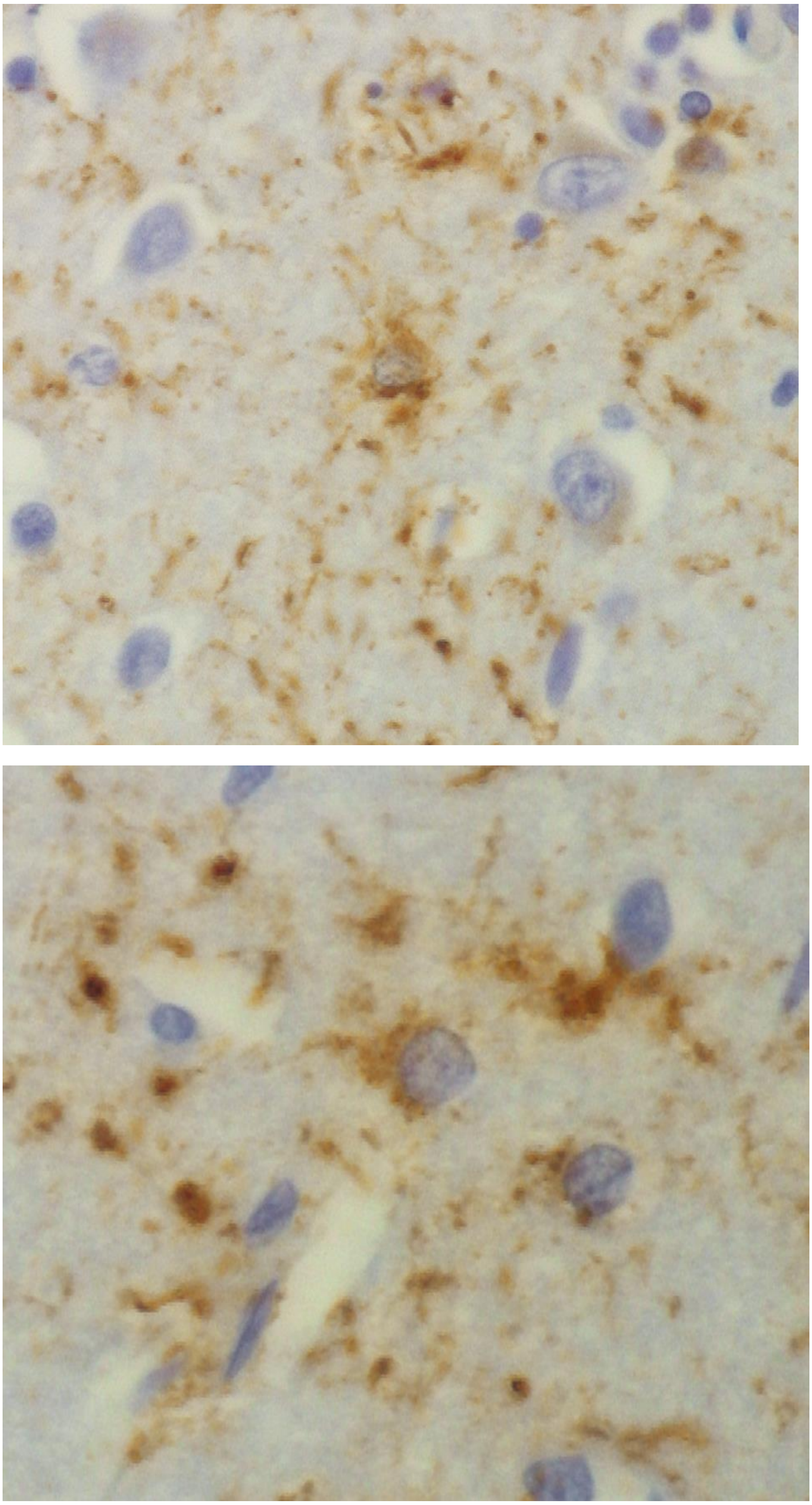

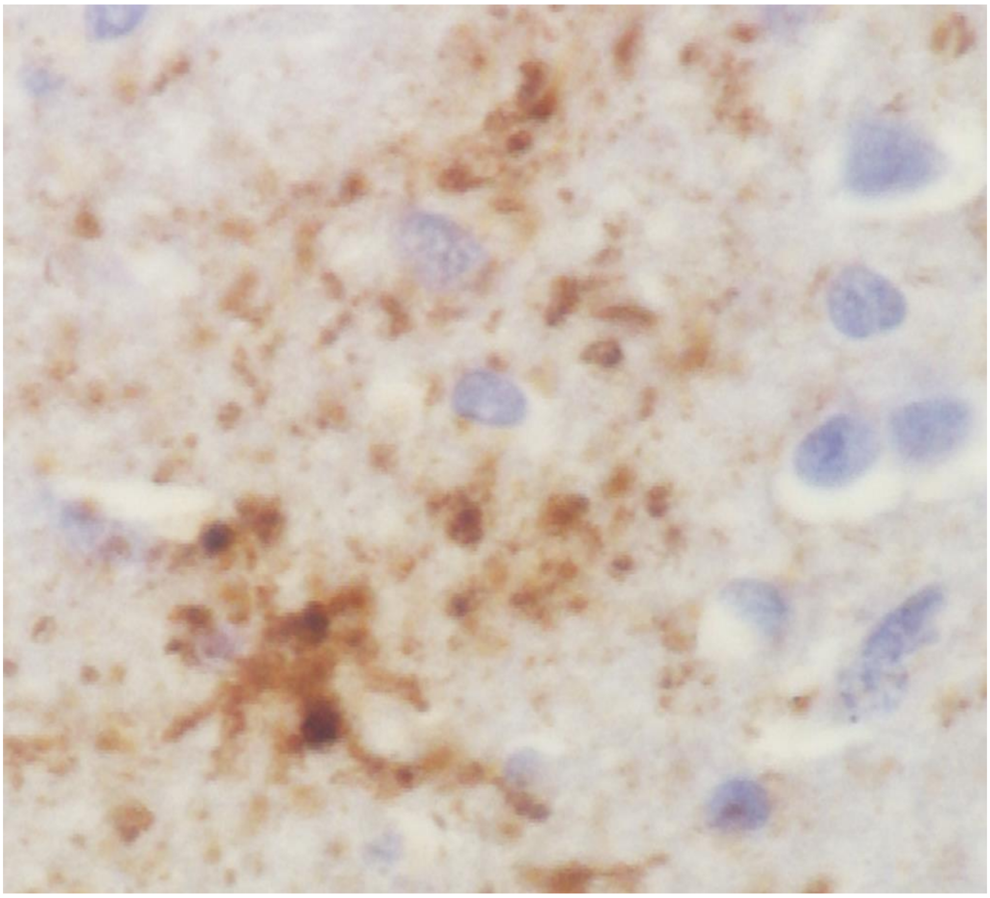

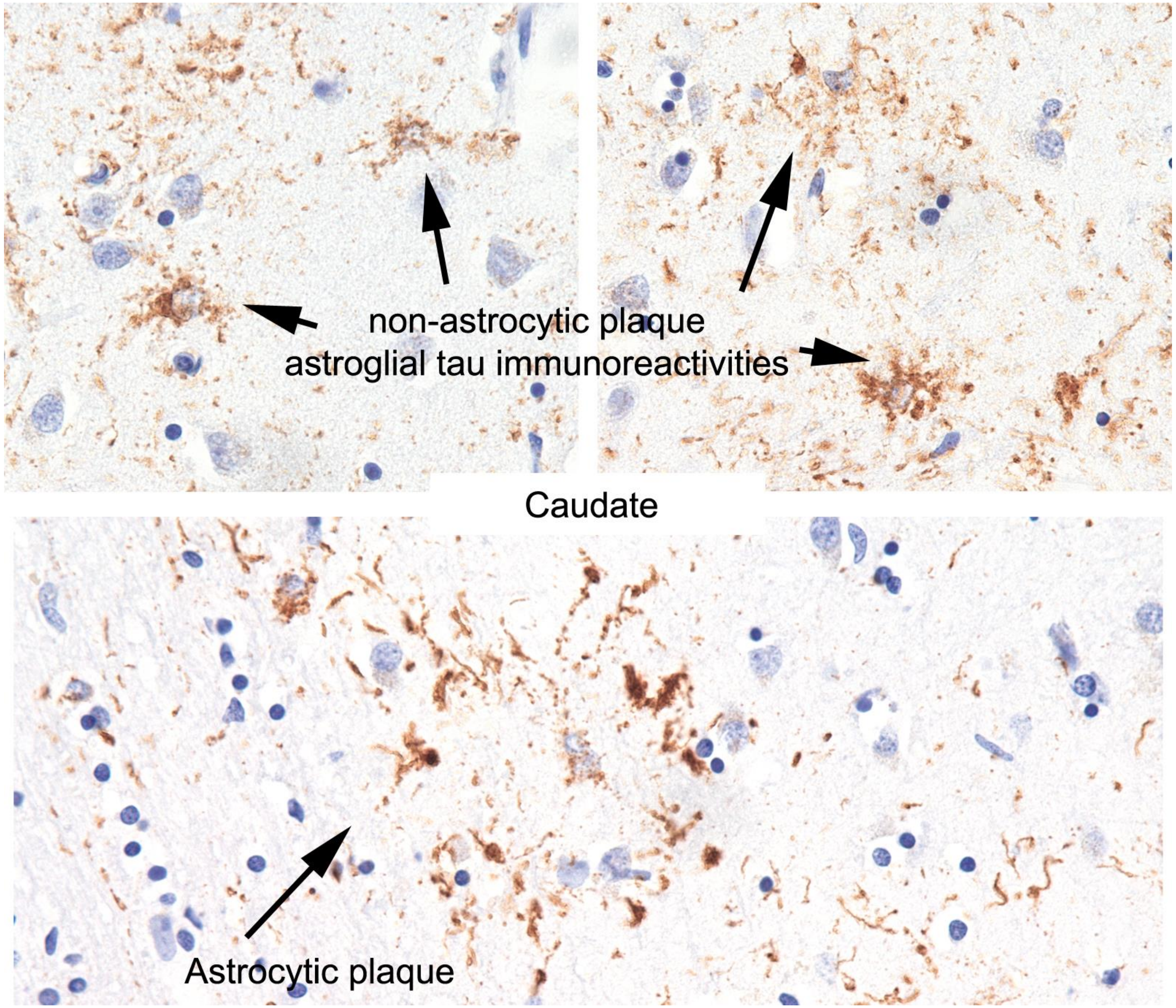

non-astrocytic plaque
astroglial tau immunoreactivities
Caudate
Astrocytic plaque

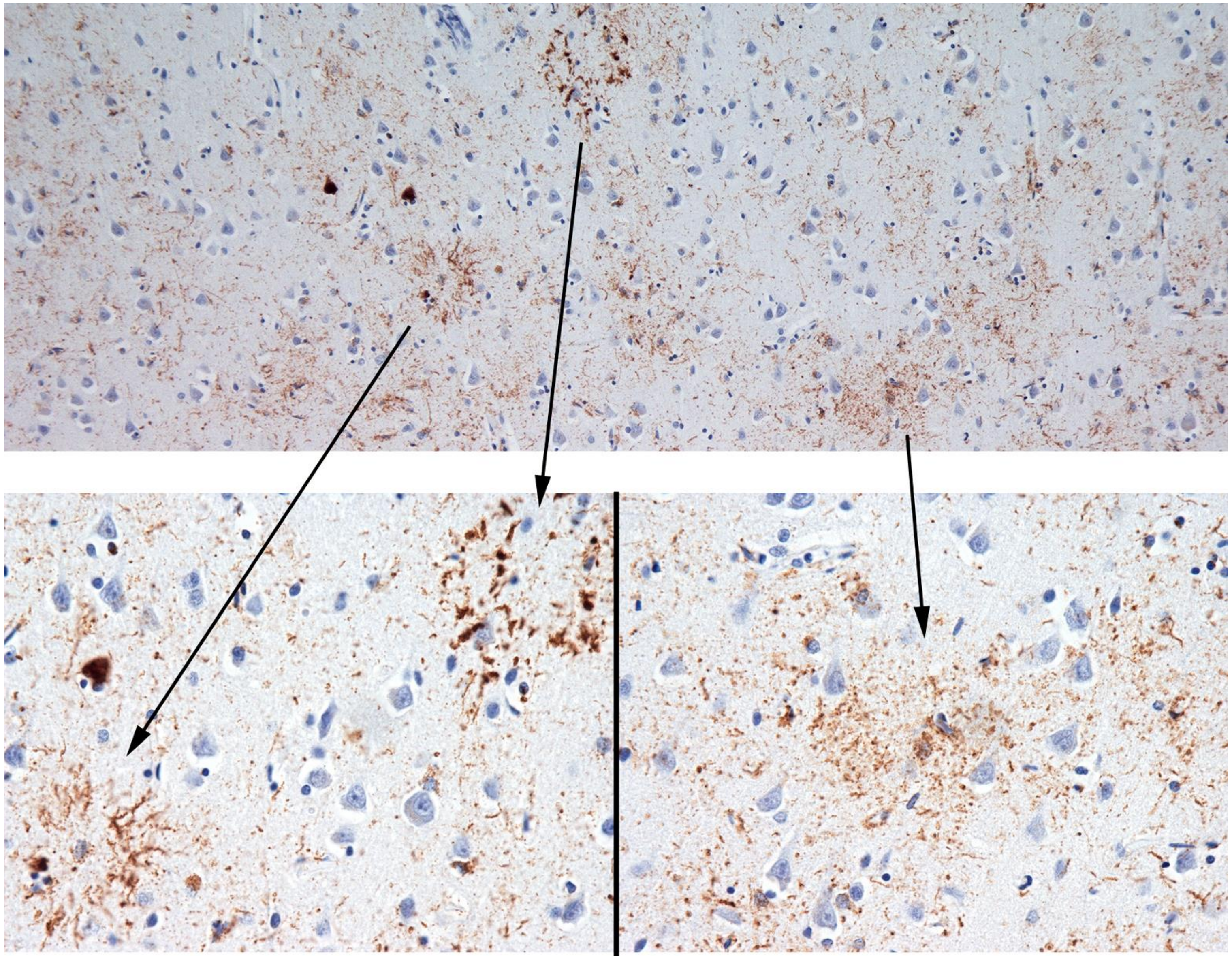

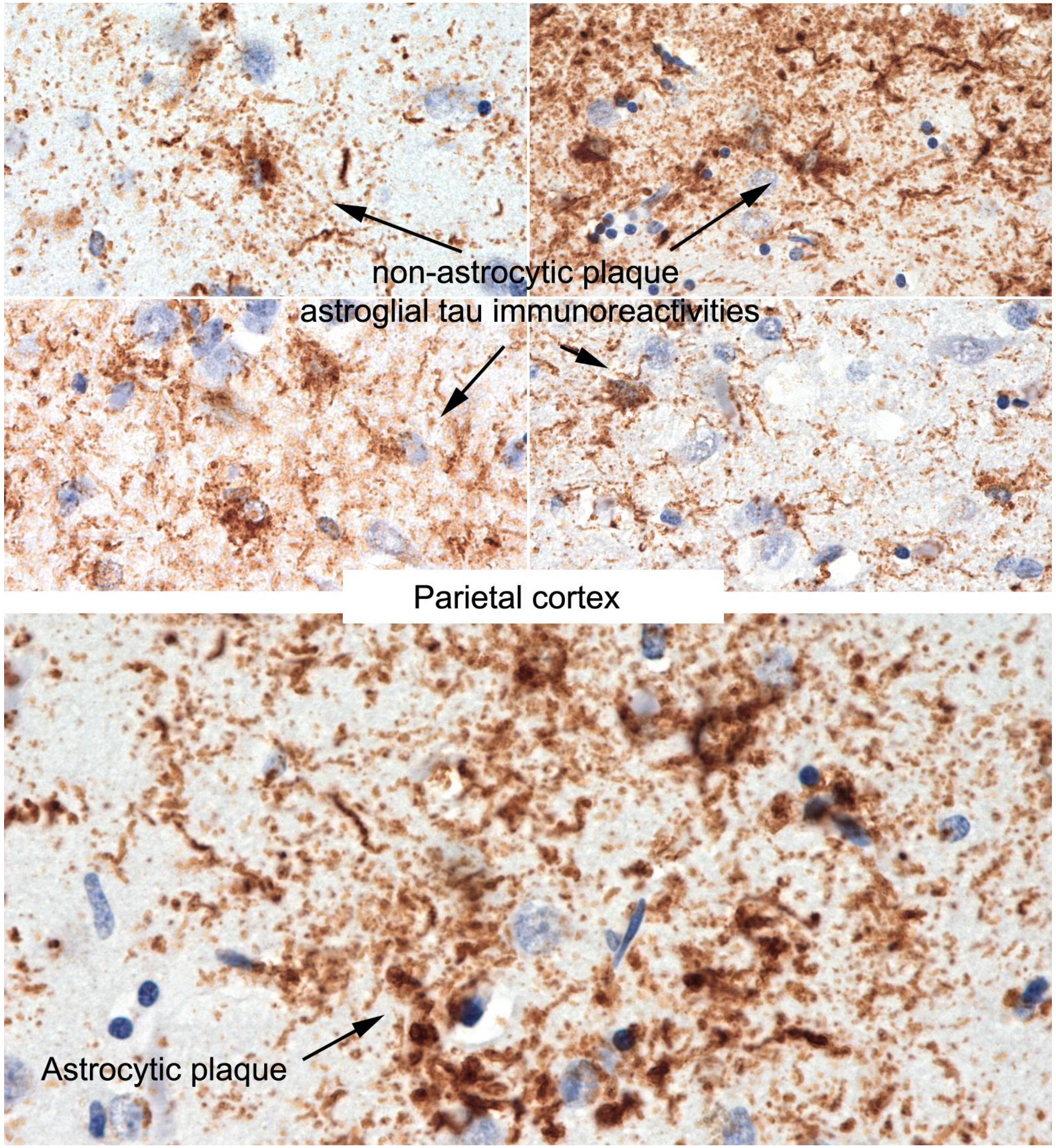
non-astrocytic plaque
astroglial tau immunoreactivities
Parietal cortex
Astrocytic plaque
