# Supplementary material for: Sequential stages and distribution patterns of aging-related tau astrogliopathy (ARTAG) in the human brain
Source: Acta Neuropathol Commun. 2018 Jun 11;6:50. doi: 10.1186/s40478-018-0552-y (PMC5996526; doi:10.1186/s40478-018-0552-y)
Supplement: Supplementary file 2 — Pairwise conditional probability matrix and odds ratios of different ARTAG types. (PDF 876 kb) [file 40478_2018_552_MOESM2_ESM.pdf]

Sequential stages and distribution patterns of aging-related tau astrogliopathy (ARTAG) in the human brain

Gabor G. Kovacs[1,2], Sharon X. Xie[3], John L. Robinson[2], Edward B. Lee[2], Douglas H. Smith[4], Theresa Schuck[2], Virginia M.-Y. Lee[2], and John Q. Trojanowski[2]

1: Institute of Neurology, Medical University of Vienna, Vienna, Austria; 2: Center for Neurodegenerative Disease Research (CNDR), Institute on Aging and Department of Pathology & Laboratory Medicine; 3: Department of Biostatistics and Epidemiology; 4: Department of Neurosurgery, Center for Brain Injury and Repair, the Perelman School of Medicine (PSOM) at the University of Pennsylvania, Philadelphia, PA, USA.

SUPPLEMENTAL FILE 2

Table 1. Pairwise conditional probability matrix and odds ratios (and 95% confidence intervals) of **subpial ARTAG** in three major areas. Reading conditional probability plot from left to right shows the estimated probability that the region on the left shows subpial ARTAG before the region on the right. Reading the plot from top to bottom shows the estimated probability that the region below shows subpial ARTAG before the region above. Odds ratios are calculated using binary logistic regression analysis corrected for age and sex. For both conditional probability and odds ratios bold and underlined indicates significant values (p<0.01) and italics a p value under 0.05. - : no p value was generated. BBR: basal brain regions, LOB: lobar, BST: brainstem.

	Region	Frequency	n	Conditional probability			Odds ratio		
PART	LOB	28	7	LOB	**0.60**	0.16	LOB	0.67 (0.02-16.3)	11.7 (0.89-152.7)
	BBR	80	20	**0.88**	BBR	**0.83**	-	BBR	0.53 (0.04-6.22)
	BST	28	7	0.40	**0.28**	BST	-	-	BST
AD	LOB	20.4	19	LOB	**0.29**	0.12	LOB	0.54 (0.16-1.78)	**6.60 (2.14-20.35)**
	BBR	81.7	76	**0.83**	BBR	**0.80**	-	BBR	1.43 (0.36-5.61)
	BST	22.6	21	0.17	**0.14**	BST	-	-	BST
PART/ AD/ other	LOB	19	34	LOB	**0.32**	0.12	LOB	*0.42 (0.17-0.99)*	**6.32 (2.74-14.56)**
	BBR	81	145	**0.84**	BBR	**0.80**	-	BBR	1.05 (0.41-2.67)
	BST	21.2	38	0.14	**0.20**	BST	-	-	BST
CBD	LOB	87.9	29	LOB	1.0	**0.84**	LOB	0	-
	BBR	72.7	24	1.0	BBR	**0.72**	-	BBR	0.95 (0.13-6.63)
	BST	24.2	8	**0**	**0.22**	BST	-	-	BST
PSP	LOB	43.8	21	LOB	0.55	0.44	LOB	0.46 (0.13-1.5)	0.87 (0.21-3.48)
	BBR	62.5	30	0.70	BBR	**0.61**	-	BBR	1.54 (0.36-6.51)
	BST	25	12	0.25	**0.22**	BST	-	-	BST
PiD	LOB	66.7	2	LOB	0.50	0.50	LOB	-	-
	BBR	33	1	0	BBR	0	-	BBR	-
	BST	33	1	0	0	BST	-	-	BST

Table 2. Pairwise conditional probability matrix and odds ratios (and 95% confidence intervals) of **subpial ARTAG** in five lobar areas. Odds ratios are calculated using binary logistic regression analysis corrected for age and sex. For both conditional probability and odds ratios bold and underlined indicates significant values (p<0.01) and italics a p value under 0.05. * indicates that no p=0.013; -: no p value was generated; FR: frontal, CI: cingular, PA: parietal, TE: temporal, OCC: occipital.

Disease	Region	Frequency	n	Conditional probability					Odds ratio				
PART	FR	57.1	4	FR	1.0	0.75	0.75	1.0	FR	0	0.53 (0.01-17.62)	0.007 (0.001-5.06)	-
	CI	14.3	1	1.0	CI	1.0	0	1.0	-	CI	0	>1000	-
	PA	28.6	2	0.5	1.0	PA	0.5	1.0	-	-	PA	0.97 (0.02-33.81)	-
	TE	42.9	3	0.66	0.66	0.66	TE	1.0	-	-	-	TE	-
	OCC	0	0	0	0	0	0	OCC	-	-	-	-	OCC
AD	FR	42.1	8	FR	1.0	0.87	0.85	1.0	FR	-	0.05 (0.003-1.10)	0.11 (0.009-1.32)	-
	CI	0	0	0	CI	0.63	0.55	1.0	-	CI	-	0	-
	PA	36.8	7	0.85	1.0	PA	0.57	1.0	-	-	PA	1.07 (0.14-7.93)	-
	TE	42.1	8	0.87	1.0	0.62	TE	1.0	-	-	-	TE	-
	OCC	21.1	4	0	0	0	0	OCC	-	-	-	-	OCC
AD/PART/other	FR	41.2	14	FR	**1.0**	0.87	0.84	1.0	FR	0	*0.16 (0.02-0.97)*	*0.13 (0.02-0.83)*	-
	CI	5.9	2	**1.0**	CI	**1.0**	**0.5**	1.0	-	CI	0	1.65 (0.08-32.2)	-
	PA	35.3	12	0.83	**1.0**	PA	0.63	1.0	-	-	PA	0.75 (0.15-3.59)	-
	TE	38.2	13	0.84	**0.92**	0.69	TE	1.0	-	-	-	TE	-
	OCC	23.5	8	0	0	0	0	OCC	-	-	-	-	+
CBD	FR	72.7	24	FR	0.36	0.08	0.45	**0.66**	FR	2.01 (0.35-11.5)	*10.49 (1.01-108.8)*	1.03 (0.19-5.37)	-
	CI	51.5	17	0.25	CI	0.11	0.41	**0.76**	-	CI	2.94 (0.37-22.97)	2.7 (0.54-13.49)	-
	PA	81.8	27	0.19	0.31	PA	0.48	**0.7**	-	-	PA	0.29 (0.02-3.11)	-
	TE	54.5	18	0.23	0.28	0.22	TE	**0.61**	-	-	-	TE	8.27 (0.86-79.05)
	OCC	24.2	8	**0**	**0**	**0**	**0.12**	OCC	-	-	-	-	OCC
PSP	FR	75	18	FR	**0.88**	**0.66***	**0.72**	**0.94**	FR	0.63 (0.04-8.56)	0.99 (013-7.05)	0.77 (0.10-5.64)	0.29 (0.01-5.74)
	CI	12.5	3	**0.33**	CI	0.66	0.66	1.0	-	CI	1.0 (0.07-13.2)	1.24 (0.09-16.47)	0
	PA	33.3	8	**0.25***	0.87	PA	0.75	1.0	-	-	PA	0.73 (0.1-5.01)	0
	TE	29.2	7	**0.28**	0.85	0.71	TE	1.0	-	-	-	TE	0
	OCC	8.3	2	**0.5**	1.0	1.0	1.0	OCC	-	-	-	-	OCC
PiD	FR	100	3	FR	0.66	0.66	1.0	1.0	FR	0	-	-	-
	CI	33.3	1	1.0	CI	0	1.0	1.0	-	CI	-	-	-
	PA	33.3	1	1.0	0	PA	1.0	1.0	-	-	PA	-	-
	TE	33.3	1	0	0	0	TE	1.0	-	-	-	TE	-
	OCC	33.3.	1	0	0	0	0	OCC	-	-	-	-	OCC

Table 3. Pairwise conditional probability matrix and odds ratios (and 95% confidence intervals) generated by binary logistic regression (here without corrections for other variables) of **subependymal ARTAG** in three major areas. For both conditional probability and odds ratios bold and underlined indicates significant values ($p<0.01$) and italics a p value under 0.05. - : no p value was generated. BBR: basal brain regions, LOB: lobar, BST: brainstem.

	**Region**	**Frequency**	**n**	**Conditional probability**			**Odds ratio**		
PART	MTL	87.5	7	MTL	1.0	*0.85*	MTL	-	-
	SC	12.5	1	0	SC	0.14		SC	-
	BST	11.1	1	0	0.14	BST			BST
AD	MTL	81.8	27	MTL	**0.83**	**0.82**	MTL	*0.88 (0.11-6.89)*	0.63 (0.05-7.63)
	SC	27.3	9	**0.33**	SC	0.28		SC	0.83 (0.07-9.49)
	BST	12.5	4	**0.16**	0.13	BST			BST
AD/PART/other	MTL	81.5	53	MTL	**0.88**	**0.82**	MTL	*0.24 (0.06-0.98)*	0.44 (0.07-2.84)
	SC	23.1	15	**0.45**	SC	0.24		SC	0.57 (0.06-5.46)
	BST	9.4	6	**0.16**	0.10	BST			BST
CBD	MTL	90.9	10	MTL	1.0	0.90	MTL	0	-
	SC	40	4	1.0	SC	0.4		SC	-
	BST	0	0	0	0	BST			BST
PSP	MTL	70	14	MTL	*0.71*	**0.73**	MTL	-	0
	SC	22.2	4	*0.33*	SC	0.23		SC	0
	BST	5	1	**0.16**	0.07	BST			BST
PiD	MTL	100	1	MTL	-	-	MTL	-	-
	SC	100	1	-	SC	-		SC	-
	BST	100	1	-	-	BST			BST

Table 4. Pairwise conditional probability matrix and odds ratios (and 95% confidence intervals) of **white matter ARTAG** in three major areas. Odds ratios are calculated using binary logistic regression analysis. Correction for age and sex and Braak stage of neurofibrillary degeneration does not change the results. For both conditional probability and odds ratios bold and underlined indicates significant values (p<0.01) and italics a p value under 0.05. - : no p value was generated. MTL: medial temporal lobe, LOB: lobar, BST: brainstem.

	Region	Frequency	n	Conditional probability			Odds ratio		
PART	LOB	12.5	2	LOB	**0.33**	0.07	LOB	0.16 (0.007-3.89)	13 (0.41-404)
	MTL	81.3	13	**0.85**	MTL	**0.78**	-	MTL	>4000
	BST	12.5	2	0.07	**0**	BST	-	-	BST
AD	LOB	48.7	55	LOB	**0.64**	**0.5**	LOB	*0.38 (0.16-0.89)*	0.88 (0.31-2.50)
	MTL	69	78	**0.78**	MTL	**0.69**	-	MTL	1.07 (0.34-3.31)
	BST	15	17	**0.16**	**0.14**	BST	-	-	BST
AD/ PART/ other	LOB	33.7	63	LOB	**0.49**	**0.34**	LOB	**0.38 (0.20-0.75)**	0.84 (0.37-1.92)
	MTL	71.1	133	**0.78**	MTL	**0.70**	-	MTL	1.26 (0.52-3.02)
	BST	17.1	32	**0.18**	**0.15**	BST	-	-	BST
CBD	LOB	42.9	9	LOB	0.60	**0.40**	LOB	0.40 (0.05-3.12)	>2400
	MTL	76.2	16	0.85	MTL	**0.75**	-	MTL	>5000
	BST	4.8	1	**0**	**0**	BST	-	-	BST
PSP	LOB	23.8	10	LOB	**0.50**	0.25	LOB	0.24 (0.40-1.46)	0.75 (0.07-7.61)
	MTL	85.7	36	**0.90**	MTL	**0.86**	-	MTL	0.64 (0.05-7.01)
	BST	11.9	5	0.12	**0.16**	BST	-	-	BST
PiD	LOB	0	0	LOB	0	0	LOB	-	-
	MTL	100	2	1.0	MTL	1.0	-	MTL	-
	BST	50	1	0.5	0.5	BST	-	-	BST

Table 5. Pairwise conditional probability matrix and odds ratios (and 95% confidence intervals) of **white matter ARTAG** in five lobar areas in Alzheimer diseased cases. Odds ratios are calculated using binary logistic regression analysis. Correction for age and sex and Braak stage of neurofibrillary degeneration does not change the results. Bold and underlined indicates significant values ($p<0.01$). FR: frontal, CI: cingular, PA: parietal, TE: temporal, OCC: occipital.

FR	0.37	0.29	0.39	**0.35**
0.29	CI	0.37	0.32	**0.31**
0.38	0.52	PA	0.64	**0.50**
0.54	0.51	0.65	TE	**0.51**
**0**	**0.03**	**0.04**	**0**	OCC

FR	1.69 (0.53-5.34)	2.77 (0.89-8.57)	1.09 (.35-3.35)	>2000
-	CI	0.805 (0.26-2.45)	1.29 (0.42-4.00)	4.28 (0.35-51.32)
-	-	PA	0.39 (0.13-1.19)	2.00 (0.16-23.69)
-	-	-	TE	>1500
-	-	-	-	OCC

Table 6. Pairwise conditional probability matrix and odds ratios (and 95% confidence intervals) of **gray matter ARTAG** in four major anatomical areas. Odds ratios are calculated using binary logistic regression analysis. Correction for age and sex and Braak stage of neurofibrillary degeneration and presence of grains in the medial temporal lobe does not change the results. For both conditional probability and odds ratios bold and underlined indicates significant values (p<0.01) and italics a p value under 0.05. - : no p value was generated.

MTL: medial temporal lobe, LOB: lobar, SC: Subcortical, BST: brainstem.

* correction only for MTL grains does not change significance but correction for all four variables (age, sex, Braak stage, grains) associates with p=0.5; §: significance lost after corrections; §§ remains significant after correct for sex, and for sex and age but p= 0.06 after additional correction for Braak stage of neurofibrillary degeneration.

	Region	Frequency	n	Conditional probability				Odds ratio			
PART	MTL	77.4	24	MTL	**0.66**	**0.77**	**0.87**	MTL	-	1.02 (0.16-6.62)	*0.10 (0.01-0.73)§*
	LOB	32.3	10	**0**	LOB	0.18	0.29	-	LOB	*9.00 (1.55-52.26)§§*	1.82 (0.32-10.34)
	SC	29.0	9	**0.28**	0.14	SC	0.22	-	-	SC	0.97 (0.15-6.24)
	BST	22.6	7	**0.57**	0.19	0.29	BST	-	-	-	BST
AD	MTL	50.0	32	MTL	0.41	0.69	*0.56*	MTL	*3.02 (1.01-9.00)*	*0.27 (0.09-0.78)§*	0.44 (0.14-1.34)
	LOB	34.4	22	0.22	LOB	**0.23**	0.40	-	LOB	2.42 (0.79-7.40)	0.40 (.11-1.40)
	SC	59.4	38	0.74	**0.52**	SC	**0.57**	-	-	SC	1.25 (0.41-3.77)
	BST	29.7	19	*0.38*	0.35	**0.26**	BST	-	-	-	BST
AD/ PART/ other	MTL	71.0	115	MTL	**0.68**	**0.81**	**0.79**	MTL	1.63 (0.74-3.56)	**0.27 (0.13-.055)***	**0.16 (0.07-0.38)**
	LOB	30.9	50	**0.23**	LOB	0.21	*0.31*	-	LOB	**3.64 (1.80-7.33)**	0.88 (0.37-2.09)
	SC	35.2	57	**0.56**	0.25	SC	**0.32**	-	-	SC	1.96 (0.88-4.34)
	BST	19.1	31	**0.41**	*0.19*	**0.15**	BST	-	-	-	BST
CBD	MTL	92.5	37	MTL	1.0	1.0	**0.91**	MTL	0	0	-
	LOB	95.0	38	1.0	LOB	1.0	**0.94**	-	LOB	-	-
	SC	92.5	37	1.0	1.0	SC	**0.94**	-	-	SC	-
	BST	15.0	6	**0**	**0**	**0**	BST	-	-	-	BST
PSP	MTL	80.6	75	MTL	0.50	**0.80**	**0.72**	MTL	**5.75 (1.58-20.82)**	1.02 (0.10-9.81)	2.30 (0.81-6.56)
	LOB	87.1	81	0.66	LOB	0.80	**0.72**	-	LOB	1.72 (0.1716.89)	**10.38 (2.12-50.85)**
	SC	93.5	87	**0.94**	0.97	SC	**0.88**	-	-	SC	7.09 (0.75-66.33)
	BST	60.2	56	**0.44**	**0.16**	**0.20**	BST	-	-	-	BST
PiD	MTL	25.0	4	MTL	*0.25*	0.16	0.16	MTL	1.12 (0.08-15.50)	2.50 (0.19-32.19)	10.00 (0.58-171.20)
	LOB	75.0	12	*0.72*	LOB	0.83	**0.76**	-	LOB	0.40 (0.03-5.15)	0.60 (0.03-9.15)
	SC	56.3	9	0.54	0.75	SC	0.58	-	-	SC	1.42 (0.1-20.43)
	BST	18.8	3	0.09	**0.25**	0.16	BST	-	-	-	BST
