# Supplementary material for: Sequential stages and distribution patterns of aging-related tau astrogliopathy (ARTAG) in the human brain
Source: Acta Neuropathol Commun. 2018 Jun 11;6:50. doi: 10.1186/s40478-018-0552-y (PMC5996526; doi:10.1186/s40478-018-0552-y)
Supplement: Supplementary file 3 — Pairwise conditional probability matrix and odds ratios of grey matter ARTAG and primary FTLD-tauopathy associated astroglial tau immunoreactivities. (PDF 437 kb) [file 40478_2018_552_MOESM3_ESM.pdf]

Sequential stages and distribution patterns of aging-related tau astrogliopathy (ARTAG) in the human brain

Gabor G. Kovacs[1,2], Sharon X. Xie[3], John L. Robinson[2], Edward B. Lee[2], Douglas H. Smith[4], Theresa Schuck[2], Virginia M.-Y. Lee[2], and John Q. Trojanowski[2]

1: Institute of Neurology, Medical University of Vienna, Vienna, Austria; 2: Center for Neurodegenerative Disease Research (CNDR), Institute on Aging and Department of Pathology & Laboratory Medicine; 3: Department of Biostatistics and Epidemiology; 4: Department of Neurosurgery, Center for Brain Injury and Repair, the Perelman School of Medicine (PSOM) at the University of Pennsylvania, Philadelphia, PA, USA.

SUPPLEMENTAL FILE 3

Table 1. Pairwise conditional probability matrix of **gray matter ARTAG** in nine major areas. Bold and underlined indicates significant values (p<0.01) and italics a p value under 0.05.

		**Frequency**	**n**									
CBD	Frontal	95	38	Frontal	0	0.75	**0.94**	**1.0**	1.0	**1.0**	**0.97**	**0.93**
	Parietal	95	38	0	Parietal	0.75	**0.94**	**1.0**	1.0	**1.0**	**0.97**	**0.97**
	Temporal	90	36	0	0	Temporal	**0.89**	**0.88**	1.0	**0.93**	**0.88**	**0.87**
	Occipital	52.5	21	**0**	**0**	**0.50**	Occipital	0.57	**0.50**	**0.53**	**0.48**	**0.48**
	Amygdala	35	14	**1.0**	**0**	**0.25**	0.42	Amygdala	**0.50**	*0.26*	**0.34**	*0.30*
	Striatum	92.5	37	1.0	1.0	1.0	**0.94**	**0.96**	Striatum	**0.96**	**0.97**	**0.97**
	Substantia nigra	15	6	**1.0**	**1.0**	**0.33**	**0.17**	*0.04*	**0**	Sub nigra	0.15	0.12
	Pons	2.5	1	**0**	**0**	**0**	**0.05**	**0**	**0**	0	Pons	0
	Medulla oblongata	5	2	**0**	**0**	**0**	**0**	*0.04*	**0**	0.06	0.05	Med.Obl
		**Frequency**	**n**									
PSP	Frontal	81.7	76	Frontal	0.42	**0.58**	**0.63**	**0.81**	*0.80*	**0.81**	**0.81**	**0.80**
	Parietal	73.1	68	0.21	Parietal	*0.42*	**0.52**	**0.78**	**0.60**	**0.76**	**0.78**	**0.76**
	Temporal	67.7	63	**0.20**	*0.15*	Temporal	0.43	**0.66**	**0.60**	**0.66**	**0.69**	**0.67**
	Occipital	55.9	52	**0.12**	**0.15**	0.25	Occipital	**0.61**	**0.50**	**0.55**	**0.57**	**0.55**
	Amygdala	30.1	28	**0.26**	**0.36**	**0.28**	**0.34**	Amygdala	**0.20**	**0.24**	**0.31**	**0.30**
	Striatum	93.5	87	*0.93*	**0.89**	**0.92**	**0.94**	**0.93**	Striatum	**0.95**	**0.94**	**0.95**
	Substantia nigra	12.9	12	**0.06**	**0.05**	**0.06**	**0.07**	**0.04**	**0.20**	Sub nigra	**0.12**	0.09
	Pons	2.2	2	**0**	**0**	**0.03**	**0.02**	**0**	**0**	**0.01**	Pons	0.01
	Medulla oblongata	6.5	6	**0**	**0**	**0.06**	**0.02**	**0.05**	**0**	0.02	0.04	Med.Obl

		**Frequency**	**n**									
PiD	Frontal	31.3	5	Frontal	0.20	0.16	0.25	0.44	0.33	0.30	0.35	0.25
	Parietal	56.3	9	0.55	Parietal	0.60	0.57	0.66	0.66	*0.66*	*0.69*	**0.72**
	Temporal	56.3	9	0.50	0.60	Temporal	0.50	0.62	0.33	*0.50*	*0.53*	0.45
	Occipital	50	8	0.45	0.40	0.33	Occipital	0.55	0.50	0.46	0.50	0.41
	Amygdala	43.8	7	0.54	0.40	0.50	0.50	Amygdala	0.50	0.30	0.35	0.3
	Striatum	56.3	9	0.60	0.60	0.33	0.62	0.66	Striatum	*0.53*	**0.57**	*0.58*
	Substantia nigra	18.8	3	0.18	*0.20*	*0*	0.12	0	*0*	Sub nigra	0.07	0.08
	Pons	12.5	2	0.18	*0.20*	*0*	0.12	0	**0**	0	Pons	0.08
	Medulla oblongata	12.5	2	0.10	**0**	0	0	0	*0*	0	0.08	Med.Obl
AD+PART+Other	Frontal	13.6	22	Frontal	0.08	0.08	**0.12**	**0.14**	**0.07**	0.12	*0.13*	0.14
	Parietal	13.6	22	0.07	Parietal	0.03	**0.10**	**0.13**	**0.05**	0.13	0.13	0.13
	Temporal	17.3	28	0.11	0.08	Temporal	**0.17**	**0.18**	**0.06**	0.18	**0.17**	*0.17*
	Occipital	1.9	3	**0**	**0**	**0**	Occipital	**0.01**	**0**	**0.02**	0.01	0.02
	Amygdala	30.2	49	**0.31**	**0.31**	**0.31**	**0.30**	Amygdala	0.30	**0.26**	**0.26**	**0.26**
	Striatum	35.2	57	**0.29**	**0.29**	**0.29**	**0.36**	0.34	Striatum	**0.36**	**0.33**	**0.34**
	Substantia nigra	11.1	18	0.08	0.11	0.11	**0.12**	**0.02**	**0.11**	Sub nigra	**0.06**	0.06
	Pons	4.9	8	*0.04*	0.06	**0.06**	0.04	**0**	**0.05**	**0**	Pons	0.05
	Medulla oblongata	8	13	0.08	0.09	*0.08*	0.07	**0.02**	**0.08**	0.04	0.02	Med.Obl
PART	Frontal	9.7	3	Frontal	0.03	0.04	0.09	0.14	*0*	0.11	0.10	0.10
	Parietal	9.7	3	0.03	Parietal	0.04	0.09	0.14	0.04	0.11	0.10	0.10
	Temporal	19.4	6	0.14	0.14	Temporal	0.19	0.23	0.04	0.22	0.20	0.20
	Occipital	0	0	0	0	0	Occipital	0	0	0	0	0
	Amygdala	29	9	0.33	0.33	0.33	0.30	Amygdala	0.33	*0.23*	0.27	**0.26**
	Striatum	29	9	*0.21*	0.25	0.16	0.29	0.33	Striatum	0.33	0.30	*0.27*
	Substantia nigra	9.7	3	0.11	0.11	0.12	0.10	*0*	0.14	Sub nigra	0.10	0.07
	Pons	3.2	1	0	0	0	0	0	0	0	Pons	0
	Medulla oblongata	3.2	1	0.03	0.03	0.04	0.03	**0**	*0.04*	0	0.03	Med.Obl

AD	Frontal	18.8	12	Frontal	0.12	0.12	*0.14*	0.20	**0.16**	0.17	0.20	0.18
	Parietal	17.2	11	0.08	Parietal	0.05	*0.13*	0.16	**0.03**	0.18	0.18	0.19
	Temporal	20.3	13	0.12	0.09	Temporal	**0.17**	0.20	**0.03**	0.21	0.20	0.19
	Occipital	3.1	2	*0*	*0*	**0**	Occipital	**0.02**	**0**	0.04	0.04	0.04
	Amygdala	29.7	19	0.31	0.30	0.30	**0.30**	Amygdala	**0.28**	**0.24**	**0.23**	**0.25**
	Striatum	59.4	38	**0.58**	**0.52**	**0.51**	**0.58**	**0.58**	Striatum	**0.60**	**0.57**	**0.58**
	Substantia nigra	14.1	9	0.12	0.15	0.15	0.15	**0.04**	**0.15**	Sub nigra	0.07	0.08
	Pons	7.8	5	0.09	0.10	0.10	0.08	**0**	**0.11**	0	Pons	0.05
	Medulla oblongata	7.8	5	0.06	0.09	0.06	0.06	**0**	**0.07**	0.03	0.03	Med.Obl

Table 2. Odds ratios (and 95% confidence intervals) of **gray matter ARTAG** in nine areas in the pooled cohort of AD+PART+other diseases. Odds ratios are calculated using binary logistic regression analysis with correction for age and sex and Braak stage of neurofibrillary degeneration. - indicates that odds ratios were not generated.

AD+PART+Other	Frontal	**12.00 (4.17-34.49)**	**10.53 (3.44-32.20)**	-	0.89 (0.30-2.61)	*3.61 (1.15-11.30)*	*3.55 (1.07-11.73)*	0.75 (0.08-6.87)	-
		Parietal	**38.94 (12.06-125.75)**	-	0.94 (0.34-2.64)	**6.37 (2.32-17.44)**	1.32 (0.34-5.02)	0	0
			Temporal	-	0.91 (0.37-2.26)	**8.31 (3.25-21.25)**	0.88 (0.23-3.30)	0	0.87 (0.18-4.18)
				Occipital	4.60 (0.40-52.58)	-	3.39 (0.28-39.91)	0	0
					Amygdala	1.55 (0.67-3.61)	**14.26 (3.79-53.55)**	-	**9.28 (2.41-35.73)**
						Striatum	1.12 (0.40-3.07)	0.65 (0.12-3.35)	0.85 (0.24-2.91)
							Sub nigra	-	**16.46 (4.56-59.38)**
								Pons	**31.66 (6.26-160.11)**
									Med.Obl

Table 3. Pairwise conditional probability matrix of **gray matter ARTAG** in eight areas in cases where amygdala was not involved. Bold and underlined indicates significant values (p<0.01) and italics a p value under 0.05.

		**Frequency**	**n**								
AD+PART+Other	Frontal		15	Frontal	0.09	0.08	**0.13**	**0.07**	**0.14**	0.14	**0.16**
	Parietal		14	0.08	Parietal	0.03	**0.11**	**0.05**	**0.12**	0.11	*0.13*
	Temporal		19	0.12	0.08	Temporal	**0.19**	**0.08**	**0.19**	**0.17**	**0.19**
	Occipital		1	**0**	**0**	**0**	Occipital	**0**	0.01	0.01	0.01
	Striatum		37	**0.29**	**0.29**	**0.26**	**0.36**	Striatum	**0.36**	0.33	**0.34**
	Substantia nigra		3	**0.01**	**0.02**	**0.03**	0.04	**0.03**	Sub nigra	0.03	0.03
	Pons		0	0	0.06	0	0	0	0	Pons	0
	Medulla oblongata		3	**0.03**	*0.03*	**0.03**	0.02	**0.02**	0	0.03	Med.Obl

Table 4. Pairwise conditional probability matrix of **gray matter ARTAG** in eight areas in cases where striatum was not involved. Bold and underlined indicates significant values (p<0.01).

		**Frequency**	**n**								
AD+PART+Other	Frontal			Frontal	0.06	0.06	0.05	**0.07**	0.06	0.06	0.06
	Parietal			0.05	Parietal	0.03	0.02	**0.05**	0.13	0.13	0.05
	Temporal			0.06	0.04	Temporal	0.07	**0.08**	0.08	0.07	0.07
	Occipital			0	0	0	Occipital	**0**	0	0	0
	Amygdala			**0.30**	**0.30**	**0.31**	**0.30**	Amygdala	**0.25**	**0.25**	**0.25**
	Substantia nigra			0.09	0.11	0.12	0.11	**0.03**	Sub nigra	0.05	0.05
	Pons			0.04	0.06	0.06	0.04	**0**	0	Pons	0.01
	Medulla oblongata			0.07	0.09	0.09	0.10	**0.02**	0.03	0.04	Med.Obl

Table 5. Pairwise conditional probability matrix of **primary FTLD-tauopathy associated astroglial tau immunoreactivities (tufted astrocytes, astrocytic plaques and ramified astrocytes)** in nine major areas. Bold and underlined indicates significant values (p<0.01) and italics a p value under 0.05.

		Frequency	n									
CBD	Frontal	91.3	42	Frontal	0	0	1.0	1.0	1.0	1.0	1.0	1.0
	Parietal	93.5	43	0	Parietal	1.0	**1.0**	1.0	1.0	1.0	1.0	1.0
	Temporal	89.1	41	0	0.97	Temporal	**0.95**	0.83	1.0	**0.97**	1.0	0.97
	Occipital	41.3	19	0	**0**	**0**	Occipital	**0.40**	**0**	**0.48**	0.47	0.50
	Amygdala	82.6	38	0	0	0	**0.86**	Amygdala	1.0	**1.0**	0.87	0.85
	Striatum	89.1	41	0	0	1.0	**0.95**	1.0	Striatum	**0.97**	0.97	0.97
	Substantia nigra	2.2	1	0	0	**0**	**0.05**	**0**	**0**	Sub nigra	0.02	0.02
	Pons	0	0	0	0	0	0	0	0	0	Pons	0
	Medulla oblongata	0	0	0	0	0	0	0	0	0	0	Med Obl
		**Frequency**	**n**									
PSP	Frontal	75.2	82	Frontal	0.35	**0.46**	**0.62**	**0.60**	**0**	**0.72**	**0.73**	**0.73**
	Parietal	67	73	0.14	Parietal	**0.36**	**0.55**	**0.53**	**0**	**0.66**	**0.66**	**0.65**
	Temporal	58.7	64	**0**	**0.10**	Temporal	*0.44*	0.43	**0**	**0.55**	**0.55**	**0.55**
	Occipital	41.3	45	**0.05**	**0.14**	*0.20*	Occipital	0.41	**0**	*0.72*	**0.47**	**0.47**
	Amygdala	51.4	56	**0.13**	**0.21**	0.34	0.45	Amygdala	**0**	**1.0**	**0.48**	**0.47**
	Striatum	94.5	103	**0.78**	**0.82**	**0.88**	**0.93**	**0.90**	Striatum	**0.92**	**0.94**	**0.94**
	Substantia nigra	30.3	33	**0.13**	**0.21**	**0.22**	*0.38*	**0.25**	**0**	Sub nigra	**0.22**	**0.28**
	Pons	14.7	16	**0.04**	**0.06**	**0.09**	**0.11**	**0.10**	**0**	**0.05**	Pons	0.11
	Medulla oblongata	11	12	**0.04**	**0.03**	**0.05**	**0.10**	**0.06**	**0**	**0.09**	0.07	Med Obl

		**Frequency**	**n**									
PiD	Frontal	80	20	Frontal	0.33	0.40	**0.77**	**0.72**	0.66	**0.81**	**0.85**	0.81
	Parietal	60	15	0	Parietal	0.33	**0.58**	*0.50*	0.40	**0.63**	**0.68**	0.63
	Temporal	72	18	0.25	0.42	Temporal	**0.66**	0.75	0.70	**0.81**	**0.75**	0.77
	Occipital	12	3	**0**	**0**	**0**	Occipital	0.18	**0.11**	0.15	0.10	0.15
	Amygdala	40	10	**0**	*0*	0.40	0.43	Amygdala	0.11	*0.38*	*0.42*	0.36
	Striatum	56	14	0.25	0.14	0.50	**0.55**	0.33	Striatum	**0.52**	**0.60**	0.52
	Substantia nigra	12	3	**0**	**0**	**0.33**	0.10	*0*	**0**	Sub nigra	0.14	0.09
	Pons	4	1	**0**	**0**	**0**	0	*0.08*	**0.11**	0.05	Pons	0.05
	Medulla oblongata	0	0	0	0	0	0	0	0	0	0	Med Obl

Table 6. Pairwise conditional probability matrices and logistic regressions for different types of ARTAG in the amygdala, frontal region and mesencephalon. Bold and underlined indicates significant values (p<0.01) and italics a p value under 0.05. SP: subpial, SE: subependymal, GM: gray matter, WM: white matter, PV: perivascular.

Amygdala								
n=223	SP	**0.47**	0.65	0.40	0.36	**0.59**	**0.61**	**0.80**
	**0.18**	SE	**0.27**	**0.13**	**0.13**	*0.27*	0.27	**0.20**
	0.63	**0.51**	GM	0.63	0.53	**0.41**	**0.41**	**0.20**
	0.46	**0.50**	0.69	WM	**0.26**	**0.64**	**0.62**	**0.40**
	0.27	**0.36**	0.49	**0.06**	PV	**0.52**	**0.52**	**0.60**
	**0.27**	*0.18*	**0.009**	**0.29**	**0.25**	Grains	*0.03*	**0**
	**0.35**	0.23	**0.07**	**0.30**	**0.30**	*0.09*	OG	**0**
	**0.99**	**0.97**	**0.96**	**0.96**	**0.98**	**0.97**	**0.97**	Neuron

Frontal					
n=50	SP	0.29	0.48	0.33	**0.51**
	0.45	GM	0.76	0.58	**0.71**
	0.69	0.77	WM	**0.36**	**0.14**
	0.31	0.44	**0.08**	PV	**0**
	**0.91**	**0.92**	**0.76**	**0.80**	Neuron

Mesencephalon						
n=29	SP	**0.50**	0.92	**0.50**	**0.50**	0.50
	**0**	SE	**0.16**	0.08	0.08	**0**
	0.92	**0.61**	GM	**0.52**	**0.57**	0.50
	**0.07**	0.19	**0.15**	WM	0.04	**0**
	**0**	0.11	**0.15**	0.11	PV	**0**
	0.76	**0.76**	0.76	**0.75**	**0.76**	Neuron

Amygdala SP		Regressions coeff B	SE	Wald	p value	Exp(B)	95% CI for EXP(B)	
							lower	upper
single	AMY_GM	-1,046	0,279	14,007	0,000	0,351	0,203	0,608
multiple	AMY_GM	-0,888	0,308	8,294	0,004	0,411	0,225	0,753
	AGE	0,028	0,018	2,350	0,125	1,028	0,992	1,066
	SEX	-0,086	0,297	0,084	0,771	0,917	0,512	1,643
	BRAAK	0,100	0,090	1,244	0,265	1,105	0,927	1,318
single	AMY_WM	0,879	0,279	9,899	0,002	2,409	1,393	4,167
multiple	AMY_WM	0,709	0,293	5,865	0,015	2,032	1,145	3,607
	AGE	0,019	0,018	1,055	0,304	1,019	0,983	1,056
	SEX	0,066	0,295	0,050	0,822	1,069	0,599	1,907
	BRAAK	0,162	0,084	3,696	0,055	1,175	0,997	1,386
single	AMY_PV	1,479	0,291	25,796	0,000	4,388	2,480	7,765
multiple	AMY_PV	1,462	0,306	22,803	0,000	4,313	2,367	7,858
	AGE	0,013	0,019	0,490	0,484	1,014	0,976	1,052
	SEX	0,305	0,315	0,939	0,333	1,357	0,732	2,515
	BRAAK	0,162	0,087	3,513	0,061	1,176	0,993	1,394
single	AMY_SE	0,830	0,322	6,630	0,010	2,293	1,219	4,314
multiple	AMY_SE	0,804	0,332	5,858	0,016	2,234	1,165	4,284

	AGE	0,024	0,018	1,730	0,188	1,024	0,988	1,062
	SEX	0,091	0,297	0,094	0,759	1,095	0,613	1,959
	BRAAK	0,182	0,083	4,790	0,029	1,200	1,019	1,412
single	MTL_Grain	-1,949	0,473	16,991	0,000	0,142	0,056	0,360
multiple	MTL_Grain	-1,858	0,485	14,666	0,000	0,156	0,060	0,404
	AGE	0,032	0,019	2,940	0,086	1,033	0,995	1,071
	SEX	0,004	0,304	0,000	0,990	1,004	0,553	1,821
	BRAAK	0,131	0,086	2,308	0,129	1,140	0,963	1,350
single	AMY_OLIG	-1,653	0,377	19,171	0,000	0,192	0,091	0,401
multiple	AMY_OLIG	-1,714	0,389	19,374	0,000	0,180	0,084	0,386
	AGE	0,040	0,019	4,399	0,036	1,041	1,003	1,081
	SEX	-0,017	0,307	0,003	0,956	0,983	0,539	1,794
	BRAAK	0,156	0,086	3,337	0,068	1,169	0,989	1,383
single	AMY_NEUR	-1,321	1,126	1,376	0,241	0,267	0,029	2,426
multiple	AMY_NEUR	-2,322	1,168	3,949	0,047	0,098	0,010	0,969
	AGE	0,033	0,018	3,293	0,070	1,034	0,997	1,072
	SEX	-0,052	0,294	0,032	0,859	0,949	0,534	1,688
	BRAAK	0,240	0,085	8,041	0,005	1,272	1,077	1,501

Amygdala GM		Regressions coeff B	SE	Wald	p value	Exp(B)	95% CI for EXP(B) lower	upper
single	AMY_WM	-0,917	0,281	10,664	0,001	0,400	0,230	0,693
multiple	AMY_WM	-0,721	0,318	5,144	0,023	0,486	0,261	0,907
	AGE	0,011	0,020	0,307	0,580	1,011	0,972	1,052
	SEX	-0,503	0,317	2,510	0,113	0,605	0,325	1,127
	BRAAK	-0,494	0,095	27,234	0,000	0,610	0,507	0,735
single	AMY_PV	-0,217	0,271	0,641	0,424	0,805	0,473	1,369
multiple	AMY_PV	-0,068	0,312	0,048	0,827	0,934	0,506	1,722
	AGE	0,003	0,020	0,020	0,887	1,003	0,965	1,043
	SEX	-0,431	0,317	1,858	0,173	0,650	0,349	1,208
	BRAAK	-0,520	0,094	30,680	0,000	0,594	0,494	0,715
single	AMY_SE	-0,167	0,309	0,295	0,587	0,846	0,462	1,549
multiple	AMY_SE	-0,090	0,349	0,067	0,796	0,914	0,462	1,810
	AGE	-0,002	0,020	0,007	0,932	0,998	0,960	1,038
	SEX	-0,469	0,314	2,226	0,136	0,626	0,338	1,158
	BRAAK	-0,514	0,094	30,222	0,000	0,598	0,498	0,718
single	MTL_Grain	3,855	1,026	14,133	0,000	47,247	6,330	352,629

multiple	MTL_Grain	3,604	1,039	12,023	0,001	36,753	4,792	281,893
	AGE	-0,006	0,021	0,087	0,768	0,994	0,955	1,035
	SEX	-0,552	0,337	2,681	0,102	0,576	0,298	1,115
	BRAAK	-0,431	0,097	19,857	0,000	0,650	0,537	0,785
single	AMY_OLIG	1,952	0,417	21,886	0,000	7,042	3,109	15,954
multiple	AMY_OLIG	2,029	0,452	20,168	0,000	7,609	3,138	18,447
	AGE	-0,007	0,021	0,116	0,733	0,993	0,952	1,035
	SEX	-0,491	0,336	2,126	0,145	0,612	0,317	1,184
	BRAAK	-0,529	0,099	28,635	0,000	0,589	0,485	0,715
single	AMY_NEUR	1,433	1,126	1,618	0,203	4,190	0,461	38,106
multiple	AMY_NEUR	3,155	1,177	7,179	0,007	23,443	2,333	235,599
	AGE	-0,008	0,020	0,149	0,699	0,992	0,953	1,033
	SEX	-0,368	0,318	1,342	0,247	0,692	0,371	1,290
	BRAAK	-0,591	0,098	36,252	0,000	0,554	0,457	0,671

**Amygdala WM**		Regressions coeff B	SE	Wald	p value	Exp(B)	95% CI for EXP(B)	
							lower	upper
single	AMY_PV	3,762	0,469	64,358	0,000	43,047	17,169	107,928
multiple	AMY_PV	3,838	0,496	59,936	0,000	46,450	17,578	122,745
	AGE	0,041	0,026	2,491	0,114	1,041	0,990	1,095
	SEX	0,212	0,408	0,271	0,603	1,237	0,556	2,752
	BRAAK	0,262	0,114	5,267	0,022	1,300	1,039	1,626

single	AMY_SE	1,207	0,353	11,698	0,001	3,343	1,674	6,675
multiple	AMY_SE	1,172	0,370	10,052	0,002	3,230	1,565	6,667
	AGE	0,050	0,019	6,741	0,009	1,051	1,012	1,091
	SEX	-0,327	0,311	1,106	0,293	0,721	0,392	1,326
	BRAAK	0,243	0,088	7,645	0,006	1,275	1,073	1,514
single	MTL_Grain	-1,797	0,431	17,401	0,000	0,166	0,071	0,386
multiple	MTL_Grain	-1,766	0,453	15,213	0,000	0,171	0,070	0,415
	AGE	0,062	0,020	9,755	0,002	1,063	1,023	1,105
	SEX	-0,427	0,316	1,823	0,177	0,653	0,351	1,213
	BRAAK	0,167	0,090	3,465	0,063	1,182	0,991	1,409
single	AMY_OLIG	-0,826	0,332	6,165	0,013	0,438	0,228	0,840
multiple	AMY_OLIG	-0,897	0,353	6,450	0,011	0,408	0,204	0,815
	AGE	0,057	0,019	8,811	0,003	1,059	1,020	1,100
	SEX	-0,439	0,308	2,030	0,154	0,645	0,352	1,179
	BRAAK	0,242	0,087	7,748	0,005	1,273	1,074	1,509
single	AMY_NEUR	0,734	0,923	0,632	0,427	2,083	0,341	12,725
multiple	AMY_NEUR	-0,453	0,982	0,213	0,645	0,636	0,093	4,359

	AGE	0,052	0,019	7,556	0,006	1,053	1,015	1,093
	SEX	-0,450	0,303	2,198	0,138	0,638	0,352	1,156
	BRAAK	0,263	0,088	9,036	0,003	1,301	1,096	1,545

**Amygdala PV**		Regressions coeff B	SE	Wald	p value	Exp(B)	95% CI for EXP(B)	
							lower	upper
single	MTL_Grain	-1,857	0,505	13,518	0,000	0,156	0,058	0,420
multiple	MTL_Grain	-1,915	0,525	13,329	0,000	0,147	0,053	0,412
	AGE	0,056	0,019	8,300	0,004	1,057	1,018	1,098
	SEX	-0,851	0,314	7,347	0,007	0,427	0,231	0,790
	BRAAK	0,078	0,088	0,786	0,375	1,081	0,910	1,284
single	AMY_SE	1,515	0,337	20,169	0,000	4,551	2,349	8,816
multiple	AMY_SE	1,446	0,348	17,261	0,000	4,244	2,146	8,395
	AGE	0,044	0,019	5,186	0,023	1,045	1,006	1,086
	SEX	-0,741	0,317	5,480	0,019	0,477	0,256	0,886
	BRAAK	0,145	0,088	2,719	0,099	1,156	0,973	1,374
single	AMY_OLIG	-1,049	0,359	8,535	0,003	0,350	0,173	0,708
multiple	AMY_OLIG	-1,172	0,377	9,637	0,002	0,310	0,148	0,649
	AGE	0,055	0,019	8,320	0,004	1,057	1,018	1,098
	SEX	-0,900	0,312	8,340	0,004	0,407	0,221	0,749
	BRAAK	0,134	0,086	2,447	0,118	1,144	0,967	1,354

single	AMY_NEUR	-0,583	0,923	0,398	0,528	0,558	0,091	3,409
multiple	AMY_NEUR	-1,675	0,999	2,811	0,094	0,187	0,026	1,327
	AGE	0,052	0,019	7,385	0,007	1,053	1,015	1,093
	SEX	-0,890	0,306	8,469	0,004	0,410	0,225	0,748
	BRAAK	0,200	0,087	5,345	0,021	1,222	1,031	1,448

**Amygdala SE**		Regressions coeff B	SE	Wald	p value	Exp(B)	95% CI for EXP(B)	
							lower	upper
single	MTL_Grain	-0,848	0,510	2,762	0,097	0,428	0,157	1,164
multiple	MTL_Grain	-0,802	0,524	2,338	0,126	0,448	0,160	1,254
	AGE	0,019	0,021	0,868	0,351	1,019	0,979	1,062
	SEX	-0,585	0,340	2,952	0,086	0,557	0,286	1,086
	BRAAK	0,073	0,095	0,584	0,445	1,076	0,892	1,296
single	AMY_OLIG	-0,381	0,395	0,932	0,334	0,683	0,315	1,481
multiple	AMY_OLIG	-0,376	0,403	0,874	0,350	0,686	0,312	1,511
	AGE	0,017	0,021	0,663	0,416	1,017	0,977	1,059
	SEX	-0,638	0,339	3,542	0,060	0,529	0,272	1,027
	BRAAK	0,111	0,093	1,426	0,232	1,118	0,931	1,342
single	AMY_NEUR	0,355	1,129	0,099	0,753	1,427	0,156	13,038

multiple	AMY_NEUR	-0,123	1,181	0,011	0,917	0,884	0,087	8,941
	AGE	0,015	0,021	0,502	0,479	1,015	0,974	1,057
	SEX	-0,629	0,338	3,460	0,063	0,533	0,275	1,034
	BRAAK	0,128	0,094	1,839	0,175	1,137	0,945	1,367

**Frontal SP**		Regressions coeff B	SE	Wald	p value	Exp(B)	95% CI for EXP(B)	
							lower	upper
single	FR_GM	-0,116	0,638	0,033	0,856	0,891	0,255	3,109
multiple	FR_GM	-0,743	0,784	0,899	0,343	0,476	0,102	2,209
	AGE	0,024	0,050	0,230	0,632	1,024	0,928	1,130
	SEX	-0,215	0,743	0,084	0,772	0,807	0,188	3,459
	BRAAK	-0,363	0,239	2,301	0,129	0,696	0,435	1,112
single	FR_WM	-1,825	0,739	6,096	0,014	0,161	0,038	0,686
multiple	FR_WM	-1,775	0,810	4,799	0,028	0,170	0,035	0,830
	AGE	-0,004	0,050	0,008	0,930	0,996	0,903	1,098
	SEX	-0,159	0,779	0,041	0,839	0,853	0,186	3,925
	BRAAK	-0,038	0,253	0,022	0,882	0,963	0,586	1,583
single	FR_PV	-0,519	0,746	0,484	0,487	0,595	0,138	2,569
multiple	FR_PV	-0,182	0,821	0,049	0,825	0,834	0,167	4,168
	AGE	0,007	0,048	0,019	0,891	1,007	0,917	1,106

	SEX	-0,133	0,729	0,033	0,855	0,876	0,210	3,652
	BRAAK	-0,239	0,229	1,086	0,297	0,787	0,502	1,234
single	FR_NEUR	-1,451	0,845	2,946	0,086	0,234	0,045	1,229
multiple	FR_NEUR	-1,202	1,045	1,325	0,250	0,301	0,039	2,328
	AGE	0,006	0,048	0,016	0,899	1,006	0,916	1,105
	SEX	-0,032	0,740	0,002	0,965	0,968	0,227	4,131
	BRAAK	-0,102	0,259	0,154	0,695	0,903	0,544	1,500

**Frontal GM**		Regressions coeff B	SE	Wald	p value	Exp(B)	95% CI for EXP(B)	
							lower	upper
single	FR_WM	-2,757	0,721	14,620	0,000	0,063	0,015	0,261
multiple	FR_WM	-2,688	0,870	9,539	0,002	0,068	0,012	0,374
	AGE	0,171	0,077	4,911	0,027	1,187	1,020	1,381
	SEX	-0,510	0,900	0,321	0,571	0,600	0,103	3,503
	BRAAK	-0,599	0,297	4,077	0,043	0,549	0,307	0,983
single	FR_PV	-2,079	0,837	6,177	0,013	0,125	0,024	0,644
multiple	FR_PV	-1,711	0,954	3,215	0,073	0,181	0,028	1,173
	AGE	0,158	0,068	5,384	0,020	1,172	1,025	1,339
	SEX	-0,674	0,871	0,599	0,439	0,510	0,092	2,809
	BRAAK	-0,635	0,277	5,264	0,022	0,530	0,308	0,912
single	FR_NEUR	-1,302	0,894	2,122	0,145	0,272	0,047	1,568
multiple	FR_NEUR	0,366	1,157	0,100	0,752	1,442	0,149	13,910
	AGE	0,142	0,063	5,022	0,025	1,152	1,018	1,304
	SEX	-0,476	0,821	0,336	0,562	0,621	0,124	3,106
	BRAAK	-0,813	0,305	7,101	0,008	0,443	0,244	0,806

**Frontal WM**		Regressions coeff B	SE	Wald	p value	Exp(B)	95% CI for EXP(B) lower	upper
single	FR_PV	2,318	0,840	7,613	0,006	10,154	1,957	52,686
multiple	FR_PV	1,806	0,877	4,240	0,039	6,088	1,091	33,980
	AGE	-0,023	0,052	0,188	0,664	0,978	0,882	1,083
	SEX	-0,038	0,752	0,003	0,960	0,963	0,221	4,203
	BRAAK	0,490	0,255	3,703	0,054	1,632	0,991	2,689
single	FR_NEUR	2,079	1,124	3,421	0,064	8,000	0,883	72,449
multiple	FR_NEUR	0,872	1,297	0,452	0,501	2,392	0,188	30,378
	AGE	-0,029	0,046	0,399	0,528	0,971	0,887	1,063
	SEX	-0,150	0,696	0,047	0,829	0,860	0,220	3,367
	BRAAK	0,555	0,269	4,269	0,039	1,742	1,029	2,949

**Frontal PV**		Regressions coeff B	SE	Wald	p value	Exp(B)	95% CI for EXP(B) lower	upper
single	FR_NEUR	20,510	15191,513	0,000	0,999	807737366,945	0,000	
multiple	FR_NEUR	19,060	14849,039	0,000	0,999	189519982,961	0,000	
	AGE	-0,031	0,048	0,424	0,515	0,969	0,882	1,065
	SEX	-0,343	0,725	0,224	0,636	0,709	0,171	2,939
	BRAAK	0,632	0,359	3,102	0,078	1,881	0,931	3,799

Mesencephalon SP		Regressions coeff B	SE	Wald	p value	Exp(B)	95% CI for EXP(B) lower	upper
single	SN_GM	-22,302	11602,712	0,000	0,998	0,000	0,000	
multiple	SN_GM	-22,423	11575,222	0,000	0,998	0,000	0,000	
	AGE	-0,004	0,116	0,001	0,974	0,996	0,794	1,250
	SEX	-0,211	1,498	0,020	0,888	0,810	0,043	15,255
	BRAAK	-0,126	0,447	0,080	0,777	0,881	0,367	2,117
single	PEDUNCLE_WM	21,203	20096,485	0,000	0,999	1615474842,851	0,000	
multiple	PEDUNCLE_WM	21,846	19523,567	0,000	0,999	3073955897,650	0,000	
	AGE	-0,023	0,076	0,094	0,760	0,977	0,841	1,135
	SEX	1,925	0,957	4,044	0,044	6,852	1,050	44,706
	BRAAK	-0,162	0,325	0,248	0,618	0,850	0,449	1,609
single	MES_PV	21,123	23205,422	0,000	0,999	1491207547,247	0,000	
multiple	MES_PV	21,121	22423,315	0,000	0,999	1488114893,518	0,000	
	AGE	-0,034	0,073	0,221	0,638	0,966	0,838	1,115
	SEX	1,384	0,910	2,311	0,128	3,991	0,670	23,773
	BRAAK	-0,201	0,310	0,421	0,516	0,818	0,445	1,502
single	AQM_SE	21,123	28420,721	0,000	0,999	1491207547,247	0,000	
multiple	AQM_SE	20,761	28327,245	0,000	0,999	1038158993,725	0,000	
	AGE	0,002	0,081	0,001	0,978	1,002	0,856	1,174
	SEX	0,968	0,908	1,137	0,286	2,633	0,444	15,610
	BRAAK	-0,178	0,316	0,319	0,572	0,837	0,450	1,555

single	SN_NEUR	0,368	0,925	0,158	0,691	1,444	0,236	8,844
multiple	SN_NEUR	0,198	0,982	0,041	0,840	1,219	0,178	8,350
	AGE	-0,012	0,070	0,031	0,860	0,988	0,862	1,132
	SEX	1,340	0,869	2,380	0,123	3,821	0,696	20,977
	BRAAK	-0,197	0,305	0,418	0,518	0,821	0,452	1,492

**Mesencephalon GM**		Regressions coeff B	SE	Wald	p value	Exp(B)	95% CI for EXP(B)	
							lower	upper
single	PEDUNCLE_WM	0,932	1,225	0,578	0,447	2,538	0,230	28,021
multiple	PEDUNCLE_WM	0,436	1,342	0,106	0,745	1,547	0,111	21,476
	AGE	0,014	0,074	0,035	0,851	1,014	0,878	1,171
	SEX	-1,987	0,946	4,412	0,036	0,137	0,021	0,875
	BRAAK	0,169	0,316	0,285	0,593	1,184	0,637	2,198
single	MES_PV	19,357	16408,711	0,000	0,999	255074970,780	0,000	
multiple	MES_PV	19,340	15858,093	0,000	0,999	250792352,865	0,000	
	AGE	0,132	0,134	0,972	0,324	1,141	0,877	1,485
	SEX	0,018	1,492	0,000	0,990	1,019	0,055	18,952
	BRAAK	-0,115	0,492	0,055	0,815	0,891	0,339	2,338
single	AQM_SE	-19,699	10048,243	0,000	0,998	0,000	0,000	
multiple	AQM_SE	-19,193	8331,703	0,000	0,998	0,000	0,000	
	AGE	0,070	0,150	0,217	0,641	1,072	0,800	1,438
	SEX	18,013	8554,169	0,000	0,998	66532033,878	0,000	
	BRAAK	0,912	0,927	0,967	0,325	2,489	0,404	15,323
single	SN_NEUR	0,368	0,925	0,158	0,691	1,444	0,236	8,844

		Regressions coeff B	SE	Wald	p value	Exp(B)	95% CI for EXP(B) lower	upper
multiple	SN_NEUR	0,962	1,111	0,749	0,387	2,617	0,296	23,106
	AGE	0,018	0,072	0,062	0,803	1,018	0,884	1,172
	SEX	-2,260	1,002	5,088	0,024	0,104	0,015	0,744
	BRAAK	0,156	0,326	0,229	0,632	1,169	0,617	2,216

**Mesencephalon WM**		Regressions coeff B	SE	Wald	p value	Exp(B)	95% CI for EXP(B)	
							lower	upper
single	MES_PV	3,135	1,430	4,811	0,028	23,000	1,396	378,898
multiple	MES_PV	21,207	10925,419	0,000	0,998	1622299881,068	0,000	
	AGE	0,075	0,181	0,173	0,678	1,078	0,756	1,538
	SEX	19,277	10925,419	0,000	0,999	235337738,216	0,000	
	BRAAK	-0,167	0,688	0,059	0,808	0,846	0,220	3,259
single	AQM_SE	-18,852	20096,485	0,000	0,999	0,000	0,000	
multiple	AQM_SE	-19,194	15654,565	0,000	0,999	0,000	0,000	
	AGE	0,153	0,191	0,641	0,423	1,165	0,802	1,693
	SEX	18,979	9211,921	0,000	0,998	174829850,504	0,000	
	BRAAK	0,866	1,002	0,746	0,388	2,376	0,333	16,943
single	SN_NEUR	19,699	16408,713	0,000	0,999	358994421,706	0,000	
multiple	SN_NEUR	20,557	14935,457	0,000	0,999	846406351,836	0,000	
	AGE	0,129	0,132	0,953	0,329	1,138	0,878	1,474
	SEX	-2,208	1,554	2,017	0,156	0,110	0,005	2,314
	BRAAK	-0,280	0,459	0,373	0,542	0,756	0,308	1,857

**Mesencephalon PV**		Regressions coeff B	SE	Wald	p value	Exp(B)	95% CI for EXP(B)	
							lower	upper
single	AQM_SE	-19,210	28420,722	0,000	0,999	0,000	0,000	
multiple	AQM_SE	-20,295	25759,238	0,000	0,999	0,000	0,000	

	AGE	0,163	0,156	1,085	0,298	1,177	0,866	1,598
	SEX	0,725	1,358	0,285	0,594	2,064	0,144	29,538
	BRAAK	-0,056	0,496	0,013	0,910	0,945	0,357	2,500
single	SN_NEUR	19,357	16408,711	0,000	0,999	255074970,780	0,000	
multiple	SN_NEUR	19,340	15858,093	0,000	0,999	250792352,865	0,000	
	AGE	0,132	0,134	0,972	0,324	1,141	0,877	1,485
	SEX	0,018	1,492	0,000	0,990	1,019	0,055	18,952
	BRAAK	-0,115	0,492	0,055	0,815	0,891	0,339	2,338

**Mesencephalon SE**		Regressions coeff B	SE	Wald	p value	Exp(B)	95% CI for EXP(B) lower	upper
single	SN_NEUR	18,952	16408,711	0,000	0,999	170049976,224	0,000	
multiple	SN_NEUR	18,229	13799,901	0,000	0,999	82548832,881	0,000	
	AGE	0,045	0,160	0,078	0,781	1,046	0,764	1,431
	SEX	18,893	9865,946	0,000	0,998	160422999,901	0,000	
	BRAAK	0,588	0,914	0,414	0,520	1,800	0,300	10,804
