# Supplementary material for: Sequential stages and distribution patterns of aging-related tau astrogliopathy (ARTAG) in the human brain
Source: Acta Neuropathol Commun. 2018 Jun 11;6:50. doi: 10.1186/s40478-018-0552-y (PMC5996526; doi:10.1186/s40478-018-0552-y)

Sequential stages and distribution patterns of aging-related tau astrogliopathy (ARTAG) in the human brain

Gabor G. Kovacs[1,2], Sharon X. Xie[3], John L. Robinson[2], Edward B. Lee[2], Douglas H. Smith[4], Theresa Schuck[2], Virginia M.-Y. Lee[2], and John Q. Trojanowski[2]

1: Institute of Neurology, Medical University of Vienna, Vienna, Austria; 2: Center for Neurodegenerative Disease Research (CNDR), Institute on Aging and Department of Pathology & Laboratory Medicine; 3: Department of Biostatistics and Epidemiology; 4: Department of Neurosurgery, Center for Brain Injury and Repair, the Perelman School of Medicine (PSOM) at the University of Pennsylvania, Philadelphia, PA, USA.

SUPPLEMENTAL FILE 4

Figure 1. Hierarchical cluster analysis of **primary astrocytic tau pathology** in PSP, CBD, Pick disease and pooled cohort of primary non-FTLD-tauopathies. LOB: lobar; SC/AM: subcortical and amygdala; BST: brainstem.

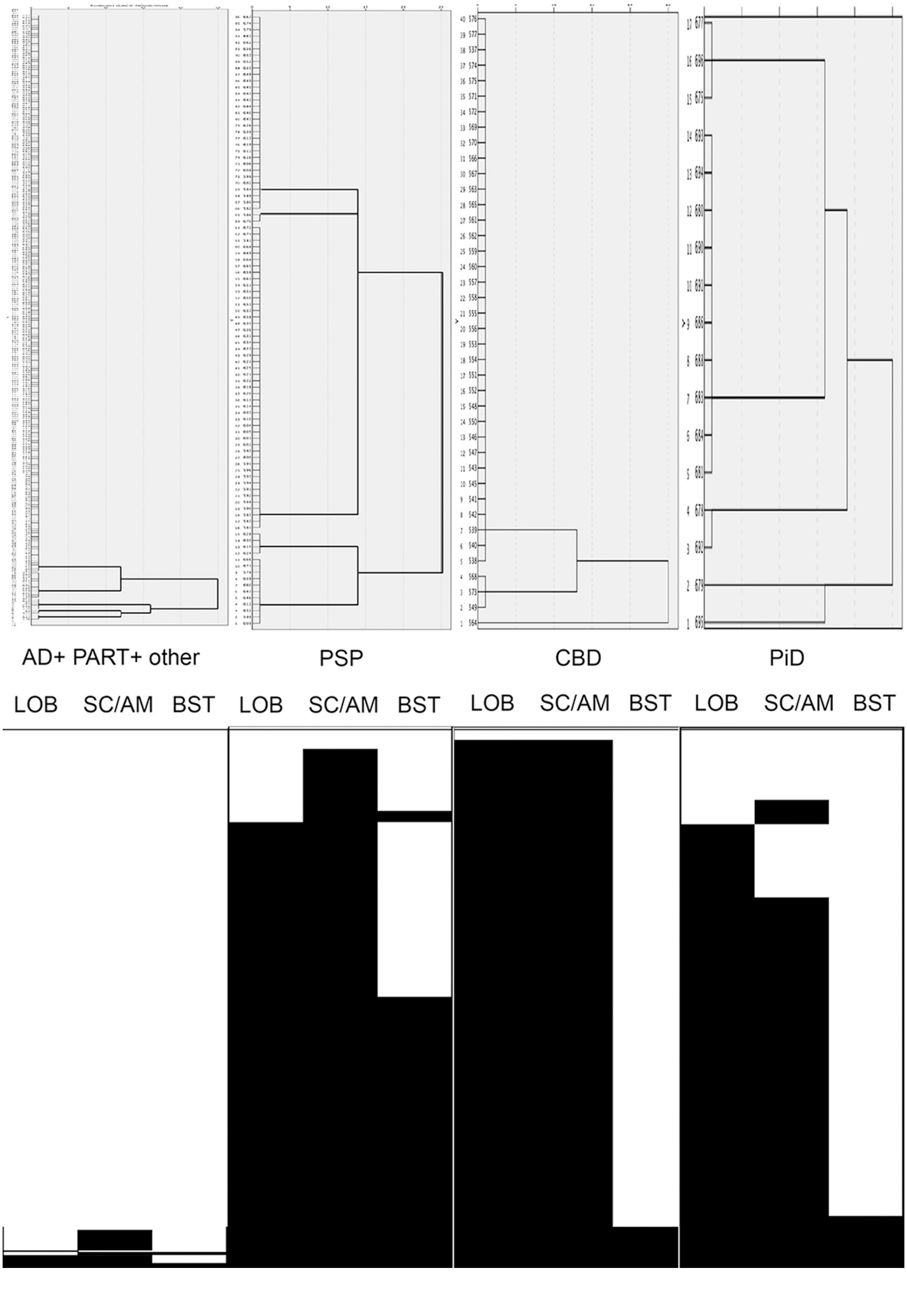

Figure 2. Hierarchical cluster analysis of **gray matter (GM) ARTAG and primary astrocytic tau pathology** (indicated as "Astro") in PSP, CBD, Pick disease and pooled cohort of primary non-FTLD-tauopathies. Lb: lobar; A/Sc: amygdala and subcortical; BST: brainstem.

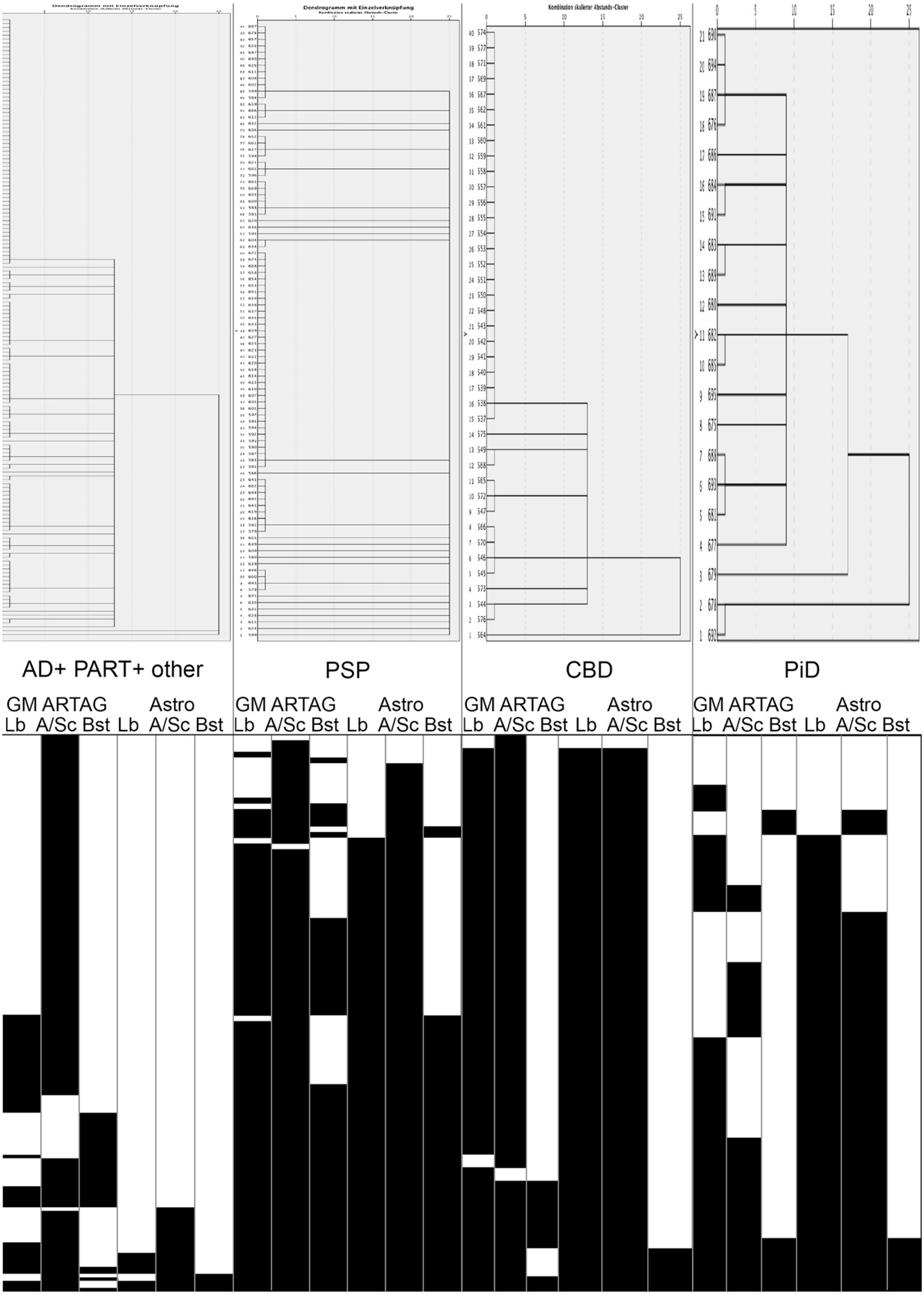

Figure 3. Hierarchical cluster analysis of subpial (SP), white (WM) and grey matter (GM) in three major regions (L: lobar; S: subcortical; B: basal brain regions including medial temporal lobe) in the pooled cohort of PART, AD and other non FTLD-tauopathies, PSP, CBD; and Pick disease. The lower part of the images shows the patterns with black boxes indicating the presence and white boxes the lack of a specific type of ARTAG in a specific region.

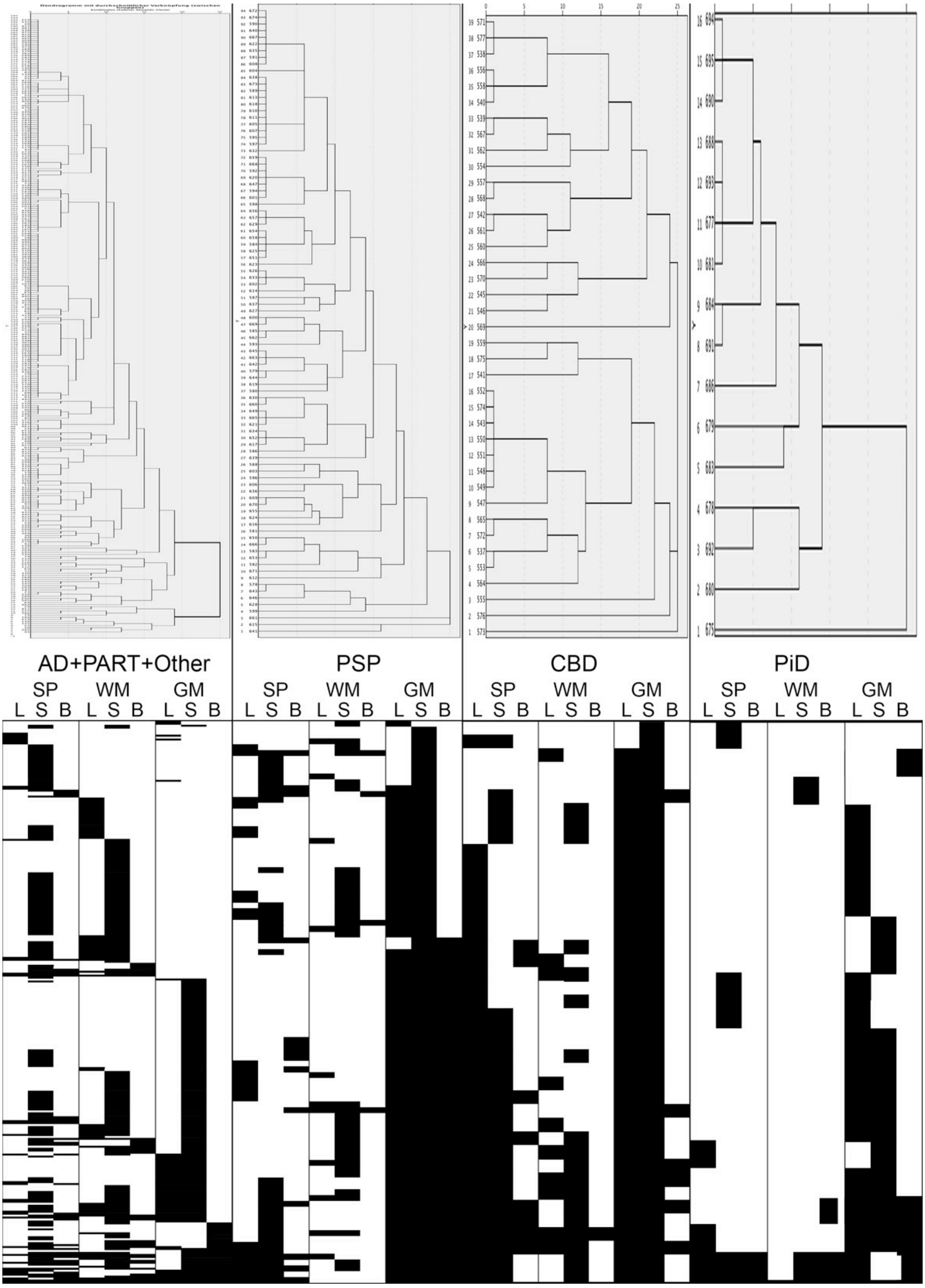

Supplement: Supplementary file 4 — Hierarchical cluster analysis of astroglial tau pathologies in different disorders. (PDF 1463 kb) [file 40478_2018_552_MOESM4_ESM.pdf]
